# Knockdown of long noncoding RNA SAN rejuvenates aged adipose-derived stem cells via miR-143-3p/ADD3 axis

**DOI:** 10.1186/s13287-023-03441-1

**Published:** 2023-08-21

**Authors:** Hewei Xiong, Sen Ren, Jing Chen, Xiaofan Yang, Yutian Liu, Zhao Xu, Jiahe Guo, Tao Jiang, Meng Yuan, Yang Liu, Guolei Zhang, Wenqing Li, Hans-Günther Machens, Zhenbing Chen

**Affiliations:** 1 Department of Hand Surgery, Union Hospital, Tongji Medical College, Huazhong University of Science and Technology, No. 1277 Jiefang Avenue, Wuhan, 430022 China; 2 Department of Emergency Surgery, Union Hospital, Tongji Medical College, Huazhong University of Science and Technology, Wuhan, 430022 China; 3 Department of Neurosurgery, Zhongnan Hospital of Wuhan University, Wuhan, 430071 China; 4 Department of Hand and Foot Surgery, Huazhong University of Science and Technology Union Shenzhen Hospital, Shenzhen, 518000 China; 5 Department of Plastic and Hand Surgery, Technical University of Munich, 81675 Munich, Germany

**Keywords:** Adipose-derived mesenchymal stem cell (ASCs), Non-coding RNA (ncRNA), Senescence, Stem cell therapy, Wound healing

## Abstract

**Background:**

Senescent adipose-derived stem cells (ASCs) exhibit reduced therapeutic efficacy during wound healing. Transcriptional regulation factors including long noncoding RNAs (lncRNAs) reportedly have essential roles in stem cell aging. However, the mechanisms of which lncRNAs influence mesenchymal stem cell aging and how it works need further investigation.

**Methods:**

The expression patterns of lncRNA senescence-associated noncoding RNA (SAN) and miR-143-3p in ASCs obtained from old and young volunteer donors were detected by quantitative polymerase chain reaction. ASCs with overexpression or knockdown of SAN and γ-adducin (ADD3) were constructed by lentiviral transduction. Mimic and inhibitor were used to manipulate the cellular level of miR-143-3p in ASCs. The effects of these RNAs on ASCs proliferation, migration and cellular senescence were examined by EdU, transwell and senescence-activated β-galactosidase (SA-β-gal) staining assays. Wound scratch and tube formation assays were conducted to evaluate the capacities of ASCs in promoting fibroblasts migration and endothelial cells angiogenesis. Furthermore, dual-luciferase assays and rescue experiments were performed to identify the RNA interactions. Finally, the therapeutic effects of SAN-depleted aged ASCs were evaluated in a skin injury model.

**Results:**

The lncRNA SAN (NONHSAT035482.2) was upregulated in aged ASCs; it controlled cellular senescence in ASCs. lncRNA SAN knockdown in ASCs led to ASC functional enhancement and the inhibition of cellular senescence; it also promoted the effects of conditioned medium (CM) on endothelial cell tube formation and fibroblast migration. Mechanistic analysis showed that SAN serves as a sponge for miR-143-3p, thereby regulating the expression of ADD3. The application of SAN-depleted aged ASCs increased re-epithelialization, collagen deposition, neovascularization and led to accelerated skin wound closure, compared with transplantation of aged ASCs.

**Conclusion:**

The lncRNA SAN mediates ASC senescence by regulating the miR-143-3p/ADD3 pathway, providing a potential target for rejuvenation of senescent ASCs and enhancement of wound repair.

**Supplementary Information:**

The online version contains supplementary material available at 10.1186/s13287-023-03441-1.

## Introduction

Adipose-derived mesenchymal stem cells (ASCs) represent promising self-renewing mesenchymal cells, which may be useful for clinical cell-based therapeutic strategies, such as wound healing [1, 2]. There is increasing evidence that the phenotypes and therapeutic properties of ASCs are negatively associated with donor age [3]; aged ASCs exhibit impaired proliferative and migrative abilities, diminished paracrine secretion, and a weakened wound healing effect [4]. Furthermore, isolated mesenchymal stem cells must be cultured in vitro, can undergo a limited number of cell divisions, and exhibit inevitable replicative senescence [5]. Elucidating the molecular mechanism of senescence in ASCs may enable the rejuvenation of cellular characteristics, thereby optimizing the effectiveness of stem cell therapy.

lncRNAs are defined as transcripts longer than 200 nucleotides [6]. Although lncRNAs have limited protein-coding potential, they have multiple roles in cellular processes [7, 8]. LncRNAs reportedly have functional roles in stem cell stemness, aged cell reprogramming, and cellular senescence [9, 10]. Previous studies have shown that Bmncr serve as a scaffold to facilitate transcriptional complex and regulates the fate of bone marrow mesenchymal stem cells during aging [11]. The lncRNA UCA1, as a direct target of repressor complex, induces senescence in primary human and mouse cells [12]. The lncRNAs MALAT1, HCG11, and SNHG5 are associated with mesenchymal stem cell differentiation [13–15]. Additionally, the lncRNA ADAMTS9-AS2 was identified as a competing endogenous RNA of miR-942-5p during the regulation of mesenchymal stem cell chondrogenic differentiation [16]. Those studies implied lncRNAs have diverse and potential roles in stem cell fate. Although many studies have focused on cellular fate or differentiation direction in stem cells, to our knowledge, the role of lncRNA in regulating of stem cell senescence has not been extensively investigated.

miRNAs, a class of small noncoding RNAs that consist of 19–23 nucleotides, can repress gene expression by binding to the 3′ untranslated region (UTR) of mRNA [17]. There is increasing evidence that miRNAs have critical roles in stem cell-related biological processes [18], including cell proliferation, migration, differentiation, and senescence [19, 20]. miR-10a and miR-155-5p were reportedly altered in aged human mesenchymal stem cells and associated with cellular functions and therapeutic outcomes of mesenchymal stem cells in the myocardial infarction model [21, 22]. Furthermore, miR-143-3p regulates myoblasts senescence, myogenesis in vitro, and muscle regeneration [23]. To our knowledge, the role of miR-143-3p in regulating ASC senescence has not been reported.

Adducin is a ubiquitous cytoskeleton protein composed of multiple subunits, which are encoded by three different genes (α-, β- and γ-adducin) [24]. γ-Adducin (ADD3) is expressed in most tissues and suggested as a novel tumor suppressor and regulates cancer cell migration, proliferation, and angiogenesis [25, 26]. The knockdown of ADD3 inhibits the expression of the p53 and p21, suggesting its functional roles in cell senescence.

In this study, we identified the lncRNA, senescence-associated noncoding RNA (SAN), as a novel regulator of ASC senescence; we showed that SAN expression is upregulated in aged ASCs. Furthermore, we explored the effects of SAN on ASC phenotype and therapeutic capacity. Mechanistic analyses indicated that SAN serves as a competing endogenous RNA for miR-143-3p, which also contributes to ASC senescence through regulation of the ADD3 gene.

## Materials and methods

### Cell isolation and culture

The isolation and culture of ASCs were previously described [27]. Subcutaneous fat was collected from volunteer donors during skin flap surgery. Human young ASCs (Y-ASCs) were obtained from young volunteer donors (8.14 ± 2.82 years of age), whereas old ASCs (O-ASCs) were obtained from old volunteer donors (54.20 ± 3.82 years of age). The basic characteristics of these donors are illustrated in Additional file 1: Table S1. Human fibroblasts and human umbilical vein endothelial cells (HUVECs) were obtained and cultured as in our previous study [28]. The isolated fibroblasts highly expressed vimentin as showed in Additional file 1: Fig. S1. This study protocol was approved by the Ethics Committee at the Tongji Medical College of Huazhong University of Science and Technology. All adult donors and the parents/guardians of all young donors provided informed consent to participate in this study.

### Cell proliferation and migration

ASCs with different treatments were trypsinized and seeded in 96-well culture plates at 6 × 10^3^ cells/well. After ASCs had been cultured overnight, they were subjected to analyses of proliferation using the BeyoClick™EDU-488 kit (C0071S, Beyotime, Shanghai, China). The relative proliferation ratio of treatment group was calculated relative to the NC group. Subsequently, lower chambers were filled with 650 µl Dulbecco’s modified Eagle medium and 3 × 10^4^ ASCs were evenly seeded in the upper chambers of Transwell plates (8.0 µm pore size, Corning, USA). After ASCs had been cultured for 24 h, cells that migrated to the lower surface of the chamber were stained with crustal violet (G1062, Solarbio, Beijing, China) and counted. The relative migration rate represents the ratio of treatment group to the NC group.

### Senescence-activated b-galactosidase (SA-β-gal) staining

ASCs (1 × 10^4^ cells/well) with different treatments were seeded in 48‐well plates. Senescent cells were stained by using an SA-β-gal staining kit (9860, Cell Signaling Technology, Boston, MA, USA), then identified via microscopy. For evaluation of H2AX, ASCs were fixed, stained with anti-H2AX antibody (0856-1-AP, Proteintech, Wuhan, China), and viewed using a fluorescence microscope. The percentages of stained cells in five random fields were calculated. The relative senescence ratio of treatment group was calculated relative to the NC group.

### HUVEC tube formation and scratch wound assay

ASCs with different treatments were cultured until they reached 80% confluence. They were then washed with phosphate-buffered saline (PBS) and incubated in serum-free medium. After 24 h of incubation, the conditioned medium (CM) was collected and centrifuged to remove nonadherent cells.

HUVECs were seeded at 2 × 10^4^ cells/well with CM in 96-well culture plates that had been coated with 70 µl Matrigel (356234, BD Biosciences, CA, USA). The formation of tubes was observed under an optical microscope after 4 h of incubation. The number of capillaries was counted, and the relative capillaries level was calculated from the ratio between treatment group and NC group.

Fibroblasts were seeded in 12-well culture plates. Cell culture medium was removed when cells reached 100% confluence; cells were then washed twice with PBS. Scratches in each well were made using a 200-µl pipette tip; ASC supernatant was then added to the wells and maintained for 24 h. Subsequently, cells were washed twice with PBS, fixed in 4% paraformaldehyde, and stained with crystal violet (G1062, Solarbio, Beijing, China). The numbers of migrated cells in the wound area were then counted under a light microscope. The relative migration rate represents the ratio of treatment group to the NC group.

### RNA sequencing analysis

The method of RNA sequencing analysis has been descripted in our previous article [27]. Briefly, clean data was got by filtering the sequence data, and then mapped to the reference genome with Bowtie2 (version 2.2.5). The noncoding transcripts were then mapped to the NONCODE database. The expected fragments per kilobase of transcript per million fragments sequenced (FPKM) was used to determine the expression level of mRNA and lncRNAs. The expression level of miRNAs was determined by the transcript per million (TPM). Differentially expressed transcripts were defined as fold changes ≥ 2 or ≤ − 2 and *q* value < 0.001 by using the DEGseq. The RNA sequencing data have been shared on the Gene Expression Omnibus (GEO) database (http://www.ncbi.nlm.nih.gov/geo; GSE174502).

### RNA isolation and quantification

Total RNA from ASCs was isolated with the miRNeasy Mini Kit (217004, Qiagen, Germany), in accordance with the manufacturer’s instructions. RNA was transcribed to cDNA using the HiScript^®^ II RT kit (R233, Vazyme, Nanjing, China) and Mir-X™ miRNA First-Strand Synthesis kit (RR638315, TaKaRa) separately. Quantitative polymerase chain reaction (qPCR) was performed using the HiScript^®^ II One Step qRT-PCR SYBR Green Kit (Q221, Vazyme). miR-143-3p RNA was reverse transcribed using a particular RT primer (Tsingke, China). The U6 and GAPDH genes were used as references for the amplification of miRNAs and other genes. Expression levels of target genes were assessed using the 2^−ΔΔCt^ method. The primer sequences are described in Additional file 1: Table S2. The RNA-seq data underlying this article have been uploaded to the Gene Expression Omnibus (http://www.ncbi.nlm.nih.gov/geo; GSE174502).

### Western blotting analysis

Lysates of treated ASCs were subjected to western blotting as previously described [28]. In brief, total protein mixtures were resolved by 10% sodium dodecyl sulfate polyacrylamide gel electrophoresis (PG01010-N, Servicebio, Wuhan, China) and then transferred to polyvinylidene difluoride membranes (IPVH00010, Millipore, USA). Membranes were incubated with primary antibodies to the following proteins: cyclin A2 (#ab181591, Abcam, USA, 1:1000), GAPDH (#10494-1-AP, Proteintech, 1:5000), fibronectin 1 (#15613-1-AP, Proteintech, 1:1000), p21 (#10355-1-AP, Proteintech, 1:1000), and ADD3 (#17585-1-AP, Proteintech, 1:1000). Membranes were then incubated with horseradish peroxidase (HRP)-labeled antibody (SA004, Aspen, China) for 1.5 h and developed using a BioSpectrum 600 Imaging System (UVP, CA, USA).

### Transfection of miR-143-3p mimic and inhibitor

An miR-43-3p mimic, inhibitor, and corresponding negative controls (mimic-NC and inhibitor-NC) were purchased from RiboBio (miR1N0000001-1-5, miR2N0000001-1-5, Guangdong, China). The sequences of these oligonucleotides are shown in Additional file 1: Table S3. Briefly, ASCs were seeded in six-well plates at 5 × 10^5^ cells/well and then transfected with 50 nM mimic (mimic-NC) or 200 nM inhibitor (inhibitor-NC) using ribo*FECT* CP Reagent (C11055-1, RiboBio) in accordance with the manufacturer’s protocol. After 48 h of incubation, ASCs were harvested for subsequent experiments.

### Dual-luciferase reporter gene assay

Seed regions of miR-143-3p binding sites in ADD3 and NONHSAT035482.2 were analyzed using biological prediction servers (RNAhybrid [https://bibiserv.cebitec.uni-bielefeld.de/rnahybrid], miRanda [http://www.microrna.org/microrna/home.do] and TargetScan [http://www.targetscan.org]). The 3′-UTR of human gene ADD3 and a corresponding mutational sequence, as well as the wild-type sequence of ADD3 and wild-type sequence of NONHSAT035482.2, were separately inserted into the siCHECK2 luciferase reporter vector (C8011, Promega, Madison, WI, USA). Lipofectamine 2000 (11668-019, Invitrogen, USA) was used to co-transfect the psiCHECK2-ADD3-3′-UTR or psiCHECK2-ADD3-3′-UTR-mut and miR-43-3p mimic or mimic-NC into HEK-293T cells that had been cultured in 24-well plates. After the cells had been cultured for 36 h, Renilla and firefly luciferase activities were measured using the Dual-Luciferase Reporter Assay System Kit (E1910, Promega).

### Viral vector construction and transduction

Lentiviral constructs for the inhibition and overexpression of ADD3 (pLKD-CMV-mCherry-puro-U6-shRNA-ADD3 and pLenti-EF1a-mCherry-P2A-puro-CMV-ADD3) and SAN (pLKD-CMV-mCherry-puro-U6-shRNA-SAN and pLenti-EF1a-mCherry-P2A-puro-CMV-SAN) were purchased from GeneChem (GeneChem, Shanghai, China), following design as previously reported [29]. The small hairpin RNA sequences of ADD3 and SAN are listed in Additional file 1: Table S4. To generate overexpression oe-ADD3 and oe-SAN constructs, the entire CDS region of ADD3 (NM_019903.5) and complete sequence of SAN were separately subcloned into the expression vector. All lentiviral constructs were able to express mCherry. ASCs at a confluence of 40–50% were incubated for 72 h with lentiviral solution that had been diluted with culture medium and polybrene (8 mg/ml).

### 5′ and 3′ rapid amplification of cDNA ends (RACE) assay

In accordance with the manufacturer’s instructions, the SMARTer RACE cDNA amplification kit (634923, Takara, CA, USA) was used to conduct 5′-RACE and 3′-RACE analyses to identify the transcriptional initiation and termination sites of SAN. Gene-specific primers used for the RACE analysis of SAN are shown in Additional file 1: Table S5.

### In vivo* wound healing model*

All animal experiments in this study were approved by the Animal Care Committee of Tongji Medical College and conducted in compliance with the ARRIVE guidelines. Male Sprague–Dawley rats (10 weeks of age) purchased from Laboratory Animal Center of Huazhong University of Science and Technology were randomly divided into four groups (*n* = 5): PBS control, Y-ASCs, O-ASCs, and sh-SAN-O-ASCs. Rats were anesthetized with intraperitoneal injections of xylazine (0.25 mg/kg) and ketamine (0.025 mg/kg), and a 1.8-cm circular diameter skin incision was made on the back of each rat; the inner skin was then removed. In total, 1.0 × 10^6^ ASCs from above groups were resuspended in 200 µl PBS, then injected subcutaneously at four points around the wound site. Photographs of wounds were acquired at 0, 3, 7, 10, and 14 days after injection; the wound area was measured in each photograph. After the sampling was finished, euthanasia of the rats was performed under anesthesia using the cervical dislocation method.

### Histological analysis

Rats were euthanized on day 14, and the skin samples were harvested, then fixed, embedded, and sectioned. To evaluate collagen accumulation in skin tissue from each rat, the sections were subjected to Masson’s staining. Hematoxylin and eosin staining was performed for histological analyses of wound repair.

### Immunofluorescence and immunohistochemistry analysis

To quantify blood vessel density, skin sections were incubated with anti-α-smooth muscle actin antibodies (ab7817, Abcam, 1:200) at 4 °C overnight. The sections were then incubated with Alexa488-conjugated secondary antibody (710,369, Invitrogen, 1:400) for 1 h at room temperature. Nuclei were stained with 4′,6-diamidino2-phenylindole (D9542-5MG, DAPI, Sigma-Aldrich). Sections were stained with DAPI alone to detect transplanted cells.

For immunohistochemistry, skin sections incubated with anti-PCNA antibody (ab29, Abcam, 1:200) at room temperature for 2 h. The sections were then incubated with HRP-conjugated secondary antibodies (A0181, Beyotime, 1:100) and visualized using diaminobenzidine. Five random fields were captured by microscope and the percent of positive cells in each field was calculated.

### Statistical analysis

Data analyses were performed with GraphPad Prism software, version 7.0. Data were presented as mean ± standard error of the mean. Unpaired Student’s *t-*tests were performed to compare data between two groups; one-way analysis of variance with Bonferroni post hoc correction was used to compare three or more groups. Pearson correlation coefficients were used to determine correlations between factors. The threshold for statistical significance was regarded as *p* < 0.05.

## Results

### Aged ASCs showed enhanced cellular senescence and impaired capacities for modulation of fibroblast and HUVEC functions

To evaluate functional alterations in ASCs during the aging process, we conducted a series of fundamental experiments. Compared with Y-ASCs, O-ASCs exhibited elevated levels of SA-β-gal activity (Additional file 1: Fig. S2a). Additionally, O-ASCs showed more DNA damage, indicated by the increased positive staining of H2AX (Additional file 1: Fig. S3a, b). Transwell migration assays showed that migration ability was impaired in O-ASCs, compared with Y-ASCs (Additional file 1: Fig. S2b). The proportion of EdU-positive cells was greater among Y-ASCs than among O-ASCs (Additional file 1: Fig. S2c), which indicated reduced proliferative capacity. Similarly, cell growth curves confirmed an inferior proliferative ability in O-ASCs, compared with Y-ASCs (Additional file 1: Fig. S3c). Neovascularization and fibroblast migration are important processes that occur during wound healing. Thus, we examined the effects of CM from Y-ASCs and O-ASCs on fibroblast migration capacity and angiogenesis. Fewer closed tubular structures and less fibroblast migration were observed in the O-ASCs-CM condition than in the Y-ASCs-CM condition (Additional file 1: Fig. S2d, e). These findings indicated that O-ASCs exhibit reduced cellular proliferation and migration, increased cellular senescence, and diminished effects on HUVEC and fibroblast functions.

### SAN knockdown reversed cellular senescence and promoted ASC proliferation, migration, and overall function

**Fig. 1:**
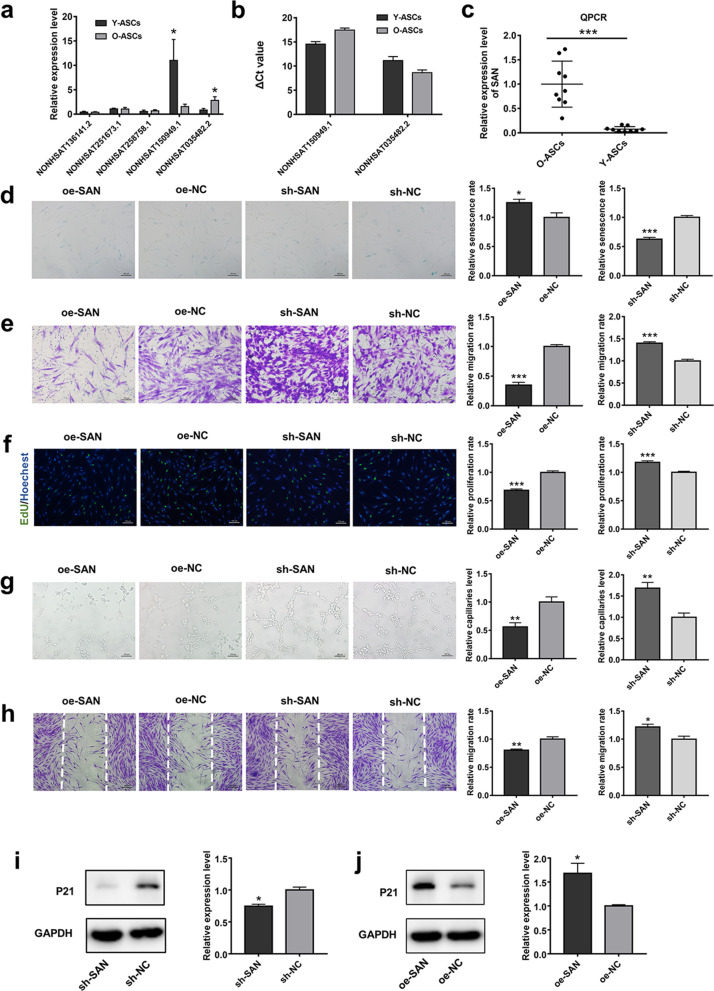
SAN expression patterns and effects of SAN in ASCs. **a** Validation of the five most altered lncRNAs by PCR analysis. *n* = 5. **b** ΔCT values of two significant altered lncRNAs. ΔCT = CT value (lncRNA)—CT value (GAPDH). *n* = 5. **c** The expression of SAN was then measured in O-ASCs and Y-ASCs. *n* = 10. **d** SA-β-gal staining and quantitative analysis of SA-β-gal-positive cells were conducted in cells subjected to SAN overexpression and knockdown. Scale bar = 100 µm. **e** Images of migrated cells and quantitative analysis of ASCs transduced with sh-NC, sh-SAN, oe-NC, or oe-SAN. Scale bar = 100 µm. **f** Representative images and quantitative analysis of EdU-stained cells (green) in ASCs that received the above treatment; nuclei were stained blue. Scale bar = 100 µm. **g** Images of tube formation and analysis of HUVECs treated with CM from ASCs in the above four groups. Scale bar = 100 µm. **h** Images and analysis of migrated fibroblasts treated with CM as described above. Scale bar = 200 µm. **i** Western blotting and quantitative analysis of the expression levels of p21 in ASCs transduced with sh-NC or sh-SAN. **j** Western blotting and quantitative analysis of the expression levels of p21 in ASCs transduced with oe-NC or oe-SAN. *n* = 3. Data are shown as the mean ± SEM. All experiments were performed in triplicate. *p < 0.05, **p < 0.01, ***p < 0.001 versus sh-NC group, #*p* < 0.05, ##*p* < 0.01, ###*p* < 0.001 versus oe-NC group. ns, not significant

To explore potential functional lncRNAs related to ASC senescence, ASCs from young and old volunteer donors were subjected to whole transcriptome resequencing. Differentially expressed lncRNAs with high fold change and low *p*-value are of considerable interest (Additional file 2). We selected the five most altered lncRNAs for further analysis. Among these lncRNAs, we found that the expression levels of NONHSAT035482.2 and NONHSAT150949.1 were significantly elevated and reduced, respectively, in qPCR analysis of O-ASCs (Fig. 1a). NONHSAT035482.2 was substantially more abundant than NONHSAT150949.1 in ASCs (Fig. 1b); therefore, we focused on NONHSAT035482.2 in subsequent experiments; we named this lncRNA “SAN.” The altered expression of SAN was confirmed on the basis of the increased sample volume (Fig. 1c). According to the NONCODE database (http://www.noncode.org/), SAN is located at chromosome 14; it has seven exons, a total length of 2,241 nucleotides, and a poly(A) tail. We performed 5′-RACE and 3′-RACE to identify the 5′-end and 3′-end cDNA sequences in SAN (Additional file 1: Fig. S4).

To elucidate the biological role of SAN in ASC senescence, lentivirus vectors were constructed carrying SAN small hairpin RNA and the whole sequence of SAN. First, knockdown efficiencies of small hairpin RNA plasmids were evaluated in 293T cells and ASCs (Additional file 1: Fig. S5a, b) and the most efficient plasmid was used in subsequent experiments. As shown in Fig. 1d, the proportion of ASCs stained with SA-β-gal was lower in the sh-SAN group than in the sh-NC group, whereas overexpression of SAN enhanced the level of SA-β-gal. Assessment of p21 protein expression also confirmed differences in cellular senescence among groups (Fig. 1i, j). The proliferation and migration capacities were significantly greater in sh-SAN ASCs than in sh-NC ASCs; in contrast, SAN overexpression led to reduced proliferation and migration (Fig. 1e, f). To investigate the role of ASCs in regulating HUVEC and fibroblast functions, which is a major component of ASC-based therapy for wound healing, we assessed the effects of CM from those four groups on angiogenesis and migration capacity. Tube formation analysis showed that HUVECs treated with CM from sh-SAN ASCs formed more tubular structures, compared with HUVECs that had been treated with CM from sh-NC ASCs. Treatment with CM from oe-SAN ASCs led to reduced endothelial network formation capacity, compared with CM from oe-NC ASCs (Fig. 1g). Furthermore, wound scratch assays showed that fibroblast migration capacity was enhanced by treatment with CM from sh-SAN ASCs, compared with CM from sh-NC ASCs; treatment with CM from oe-SAN ASCs led to diminished fibroblast migration capacity, compared with CM from oe-NC ASCs (Fig. 1h). qPCR demonstrated that mRNA expression levels of vascular endothelial growth factor A and fibroblast growth factor were significantly greater in sh-SAN ASCs than in sh-NC ASCs (Additional file 1: Fig. S5d). Collectively, these data show that SAN knockdown in ASCs can ameliorate senescence-associated phenotypes and modulate cellular functions in ASCs.

### miR-143-3p mediates cellular senescence of ASCs as a competing endogenous RNA of SAN

**Fig. 2:**
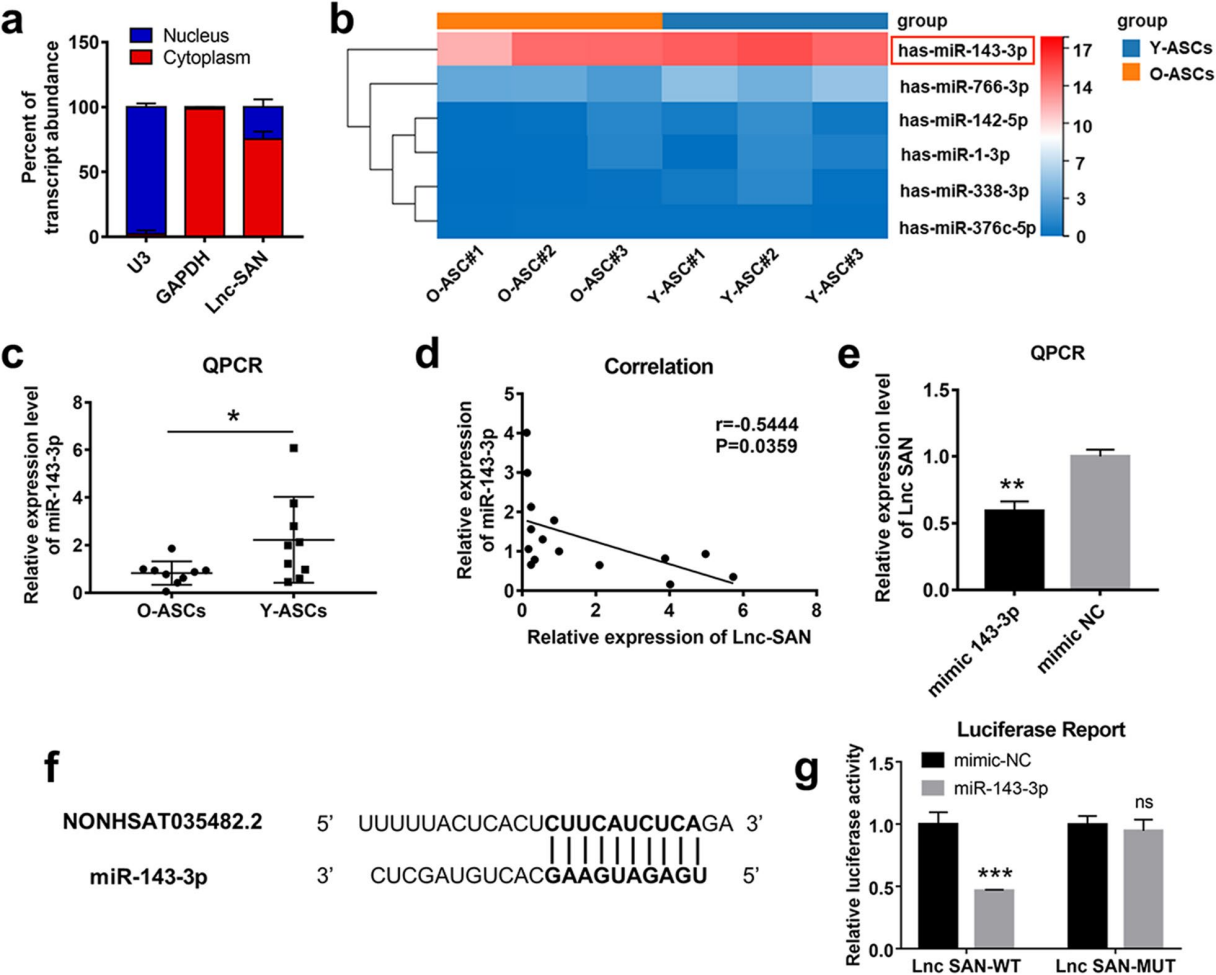
miR-143-3p mediates ASC senescence by competing with SAN. **a** qPCR was conducted to assess SAN enrichment in the nuclei and cytoplasm of ASCs; U3 and GAPDH served as respective internal controls. *n* = 3. **b** Expression profiles of miRNAs predicted to interact with SAN are shown in heatmap. **c** The expression of miR-143-3p was measured in O-ASCs and Y-ASCs by qPCR. *n* = 10. **d** Correlation analysis of the relative expression of miR-143-3p and SAN. *n* = 16. **e** qPCR assays were used to detect SAN expression in ASCs transfected with miR-143-3p mimic or mimic-NC. *n* = 3. **f** Potential binding sites of miR-143-3p and SAN. **g** Luciferase activities of the luciferase reporter gene in HEK-293 T cells co-transfected with miR-143-3p mimic or mimic-NC and with luciferase reporter vector containing SAN wild-type or MUT target sites. *n* = 3. Data are shown as the mean ± SEM. All experiments were performed in triplicate. *p < 0.05, **p < 0.01, ***p < 0.001

To further investigate the mechanism by which SAN contributes to ASC senescence, we measured the relative expression of SAN in the nucleus and cytoplasm. The results showed that SAN transcripts were primarily located in the cytoplasm (Fig. 2a). Cytoplasmic lncRNA reportedly functions as competing endogenous RNA [7]. Therefore, we screened SAN target miRNAs by means of bioinformatics prediction and sequencing data analysis. We further overlapped the targeted miRNAs of SAN and downregulated miRNAs in aged ASCs, and finally obtained six candidate miRNAs, which are clustered in Fig. 2b. Among these miRNAs, miR-143-3p exhibited the highest expression level. qPCR revealed that miR-143-3p was upregulated in Y-ASCs, compared with O-ASCs (Fig. 2c). Furthermore, Pearson correlation analysis showed a negative correlation between expression levels of SAN and miR-143-3p in ASCs (Fig. 2d). Accordingly, we proposed that SAN serves as a sponge for the regulation of miR-143-3p. To assess the direct binding of SAN and miR-143-3p, a dual-luciferase reporter gene assay was conducted, which showed that luminescence activity was significantly inhibited in the SAN-wild-type + miR-143-3p mimic group, compared with the SAN-wild-type + mimic NC group (Fig. 2f, g). Additionally, the lncRNA level was reduced after miR-143-3p overexpression, compared with the mimic NC group (Fig. 2e). Furthermore, a SAN mutant lacking the miR-143-3p binding site did not influence the proliferation, migration, or senescence of ASCs (Additional file 1: Fig. S6a–d). Overall, these findings demonstrated that SAN can target miR-143-3p and affect its function.

**Fig. 3:**
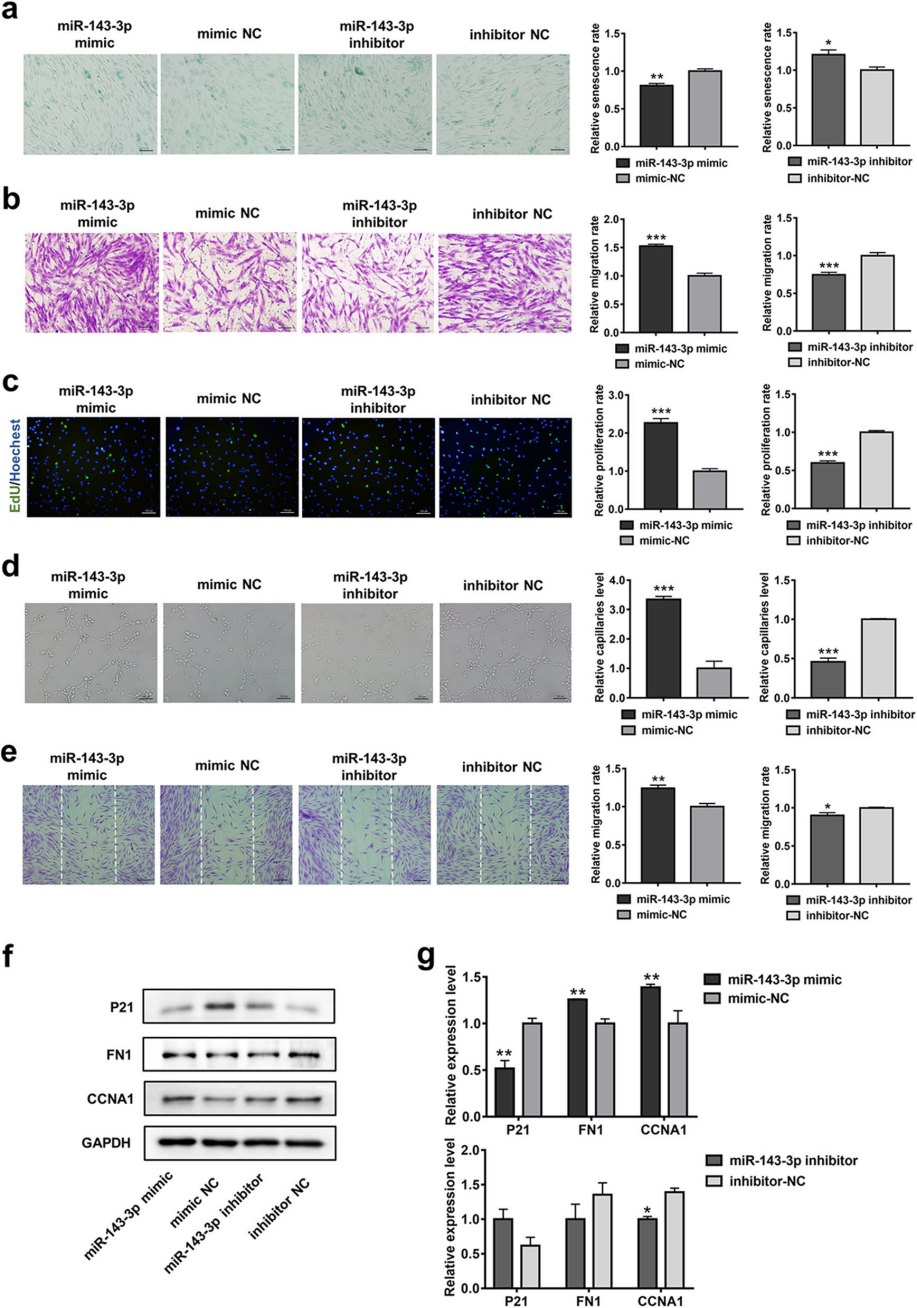
miR-143-3p mediates ASC senescence and cellular functions. **a** β-gal Staining analysis of senescent cells among ASCs treated with miR-143-3p mimic, mimic-NC, miR-143-3p inhibitor, or inhibitor-NC. Scale bar = 100 µm. **b** Representative images of migrated cells and quantitative analysis of ASCs that received the above treatment. Scale bar = 100 µm. **c** Representative images and quantitative analysis of EdU-stained cells (green) in ASCs that received the treatment; nuclei were stained blue. Scale bar = 100 µm. **d** Images of tube formation and analysis of HUVECs treated with CM from ASCs treated with miR-143-3p mimic, mimic-NC, miR-143-3p inhibitor, or inhibitor-NC. Scale bar = 100 µm. **e** Images and analysis of migrated fibroblasts treated with CM as described above. Scale bar = 200 µm. **f** Western blotting analysis of the expression levels of p21, FN1, and CCNA1 in ASCs transfected with miR-143-3p mimic, mimic-NC, miR-143-3p inhibitor, or inhibitor-NC. **g** Qualified data shown in F. Data are shown as the mean ± SEM. All experiments were performed in triplicate. *n* = 3. *p < 0.05, **p < 0.01, ***p < 0.001 versus mimic-NC group, #*p* < 0.05, ##*p* < 0.01, ###*p* < 0.001 versus inhibitor-NC group

Our findings indicated that miR-143-3p was significantly downregulated in O-ASCs. Therefore, we investigated whether miR-143-3p mediates cellular senescence in ASCs; we examined ASC proliferation, migration, senescence, and cellular function after miR-143-3p overexpression or inhibition. miR143-3p mimic, mimic-NC, miR143-3p inhibitor, or inhibitor NC was separately transfected into ASCs; transfection efficiency was evaluated by qPCR (Additional file 1: Fig. S7). Compared with the mimic-NC group, ASCs treated with miR-143-3p exhibited enhanced proliferation and migration capacities, whereas ASC proliferation and migration were reduced in the miR-143-3p inhibitor group, compared with the inhibitor-NC group (Fig. 3b, c). Additionally, overexpression of miR-143-3p reduced the number of SA-β-gal-positive cells, whereas inhibition of miR-143-3p led to larger numbers of SA-β-gal-positive cells, compared with the inhibitor-NC group (Fig. 3a). Western blotting analysis showed that treatment with miR-143-3p mimic increased the cell-cycle associated protein cyclin A1 (CCNA1) and the migration-associated protein fibronectin 1 (FN1), while reducing the expression of the senescence-associated protein p21. In contrast, treatment of ASCs with miR-143-3p inhibitor led to reduced expression levels of FN1 and CCNA1, as well as enhanced p21 expression (Fig. 3f, g). Furthermore, wound healing and tube formation assays showed that CM from the miR-143-3p-overexpressing group was more effective in modulating fibroblast activity and angiogenesis than was CM from the mimic-NC group; these effects were reduced upon treatment with CM from the miR-143-3p inhibition group, compared with the inhibitor-NC group (Fig. 3d, e). Taken together, the findings indicate that miR-143-3p acts as a cellular senescence suppressor in ASCs.

### miR-143-3p overexpression attenuated the cellular senescence effect of SAN in ASCs

**Fig. 4:**
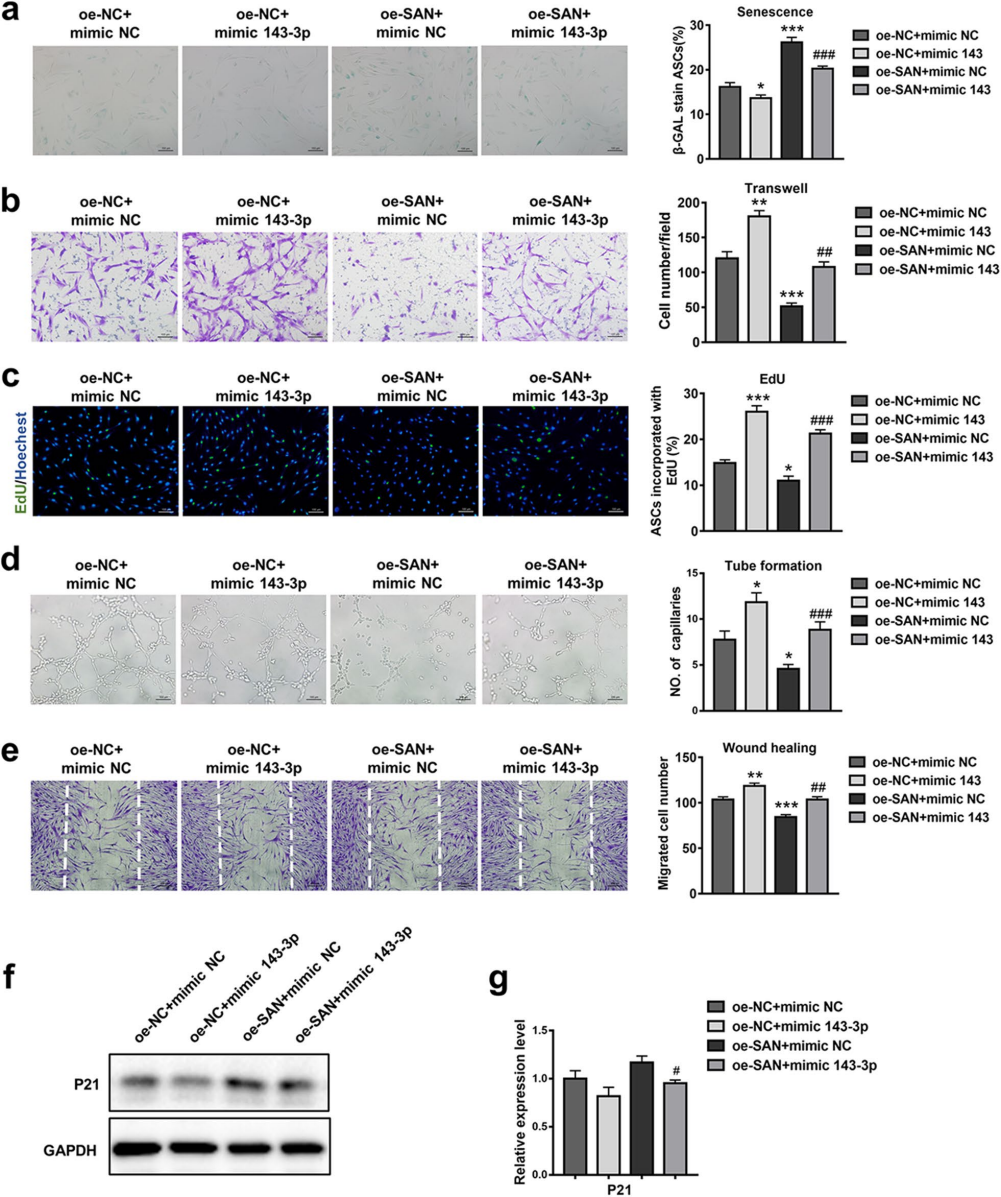
miR-143-3p overexpression attenuated the cellular senescence effect of SAN in ASCs. **a** β-gal staining and quantitative analysis of SA-β-gal-positive cells were detected in ASCs that had been stably transduced with NC vector or SAN and simultaneously transfected with miR-143-3p mimic or mimic-NC. Scale bar = 100 µm. **b** Images of migrated cells and quantitative analysis of ASCs that had been stably transduced with NC vector or SAN and simultaneously transfected with miR-143-3p mimic or mimic-NC. Scale bar = 100 µm. **c** Representative images and quantitative analysis of EdU-stained cells (green) in ASCs that received the above treatment; nuclei were stained blue. Scale bar = 100 µm. **d** Images of tube formation and analysis of HUVECs treated with CM from ASCs in the above four groups. Scale bar = 100 µm. **e** Images and analysis of migrated fibroblasts treated with CM as described above. Scale bar = 200 µm. **f** Western blotting analysis of the expression levels of p21 in ASCs that had been stably transduced with NC vector or SAN and simultaneously transfected with miR-143-3p mimic or mimic-NC. **g** Qualified data shown in f. Data are shown as the mean ± SEM. All experiments were performed in triplicate. *n* = 3. *p < 0.05, **p < 0.01, ***p < 0.001 versus oe-NC + mimic-NC group, #*p* < 0.05, ##*p* < 0.01, ###*p* < 0.001 versus oe-SAN + mimic-NC group

To detect whether the senescence-promoting effects of SAN were mediated by miR-143-3p, we treated oe-SAN ASCs with miR-143-3p mimics or mimic-NC. As shown in Fig. 4, treatment with miR-143-3p mimics rescued the inhibitory effects of oe-SAN on ASC proliferation and migration and rejuvenated the enhanced senescence phenotype in the oe-SAN group (Fig. 4a–c). Moreover, CM from oe-SAN ASCs that had been transfected with miR-143-3p exhibited restored fibroblast-modulating and angiogenic effects, compared with CM from oe-SAN ASCs that had been treated with mimic-NC (Fig. 4d, e). Western blotting analysis also indicated the restoration of p21 expression (Fig. 4f, g). Collectively, the above results showed that overexpression of miR-143-3p reversed the effects of SAN overexpression in ASCs.

### ADD3, a target gene of miR-143-3p, served as a senescence-associated gene in ASCs

**Fig. 5:**
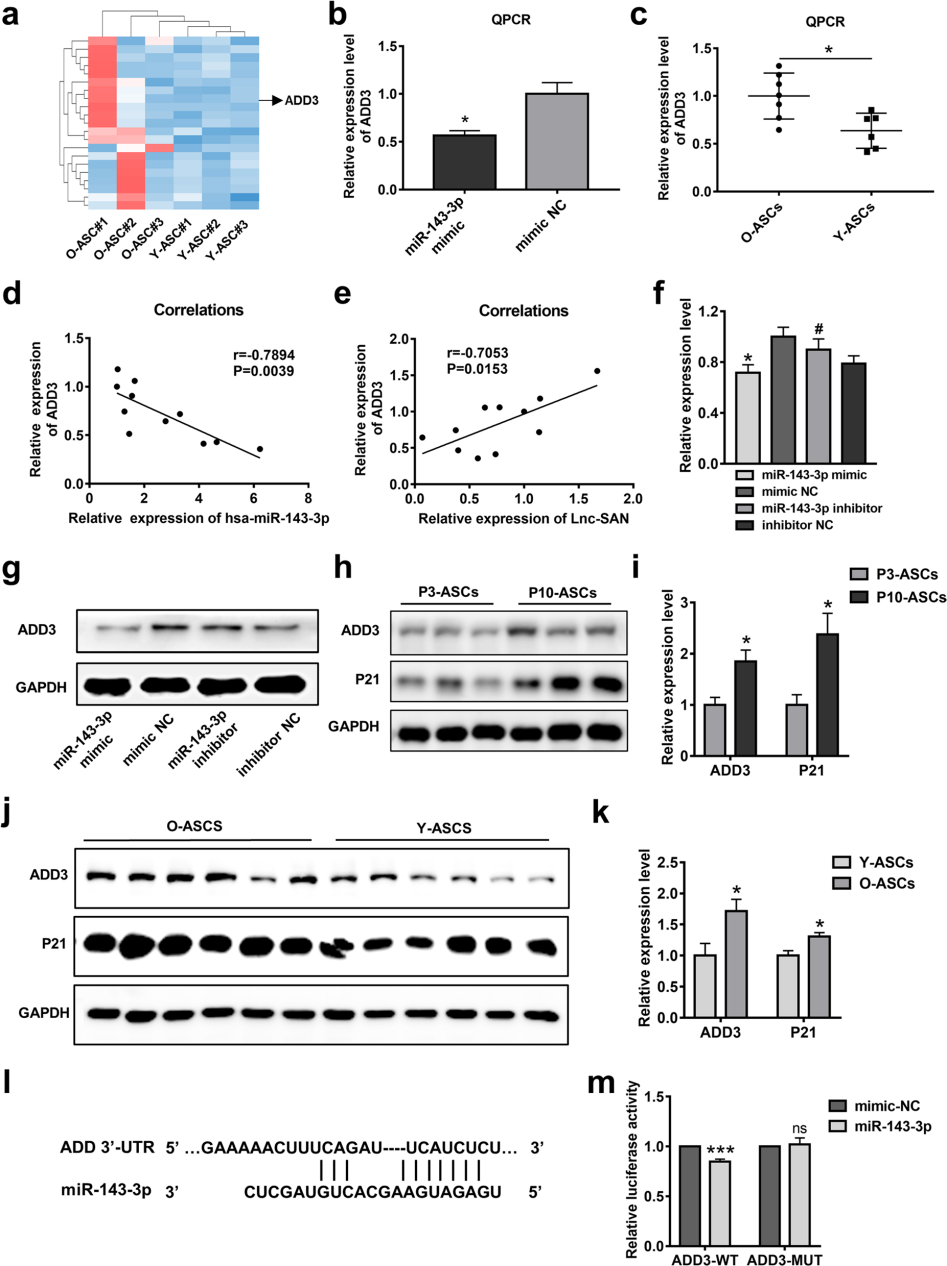
ADD3 is a target gene of miR-143-3p and acts as a senescence-associated gene in ASCs. **a** Predicted target genes of miR-143-3p are shown in the heatmap. **b** qPCR analysis of the expression levels of ADD3 in ASCs treated with miR-143-3p mimic or mimic-NC. *n* = 3. **c** qPCR analysis of the expression levels of ADD3 in O-ASCs and Y-ASCs. *n* = 10. **d**, **e** Correlation analysis of the expression levels of ADD3 with the expression levels of miR-143-3p and SAN. *n* = 11. **f** Qualified data shown in (**g**). **g** Western blotting analysis of the relative ADD3 protein levels in ASCs transfected with miR-143-3p mimic, mimic-NC, miR-143-3p inhibitor, or inhibitor-NC. **h** Western blotting analysis of the ADD3 and p21 protein levels in passage 3 ASCs and passage 10 ASCs. *n* = 3. **i** Qualified data shown in (**h**). **j** Western blotting analysis of the ADD3 and p21 protein levels in O-ASCs and Y-ASCs. **k** Qualified data shown in (**j**). **l** Potential binding sites for miR-143-3p in the 3’-UTR of ADD3. **m** Luciferase activities of HEK-293 T cells co-transfected with miR-143-3p mimic or mimic-NC, and co-transfected with luciferase reporter vector containing wild-type or MUT 3′UTR of ADD3.*n* = 6–7. All experiments were performed in triplicate. *p < 0.05, **p < 0.01, ***p < 0.001. ns, not significant

To elucidate the underlying mechanism of miR-143-3p, bioinformatics databases including TargetScan, miRanda, and RNAhybrid were used to identify potential targets of miR-143-3p. The miR-143-3p targeted genes with high expression in aged ASCs were screened out and then depicted in heatmaps (Fig. 5a). Of them, ADD3 exhibited the higher expression level, larger fold change, and lower *P*-value (Additional file 3). qPCR analysis showed that ADD3 was inhibited after the overexpression of miR-143-3p, whereas it was upregulated in O-ASCs, compared with Y-ASCs (Fig. 5b, c). Thus, ADD3 was selected for further validation. Correlation analyses revealed that the expression level of ADD3 was significantly negatively correlated with the expression level of miR-143-3p; it was significantly positively correlated with the expression level of SAN (Fig. 5d, e). To test whether miR-143-3p regulates ADD3 protein expression in ASCs, we transfected miR-143-3p mimic or miR-143-3p inhibitor into ASCs; we used mimic-NC or inhibitor-NC as control treatments. Western blotting analysis showed significantly reduced ADD3 protein expression in miR-143-3p mimic-treated ASCs, compared with the mimic-NC group. Similarly, the inhibition of miR-143-3p in ASCs led to enhanced expression of ADD3 (Fig. 5f, g). Furthermore, SAN overexpression increased the protein expression level of ADD3, whereas SAN depletion led to reduced ADD3 expression (Additional file 1: Fig. S8a, b). Luciferase reporter assays were conducted to identify the putative seed-matched sequence in the ADD3 3′-UTR region. Co-transfection of the ADD3-wild-type (WT) plasmid and miR-143-3p mimic into HEK-293T cells led to repression of luciferase activity compared with luciferase activity in the mimic-NC group, whereas no significant change was observed in the ADD3-mutant-type (MUT) group (Fig. 5l, m). These results suggested that ADD3 serves as a target gene for miR-143-3p.

**Fig. 6:**
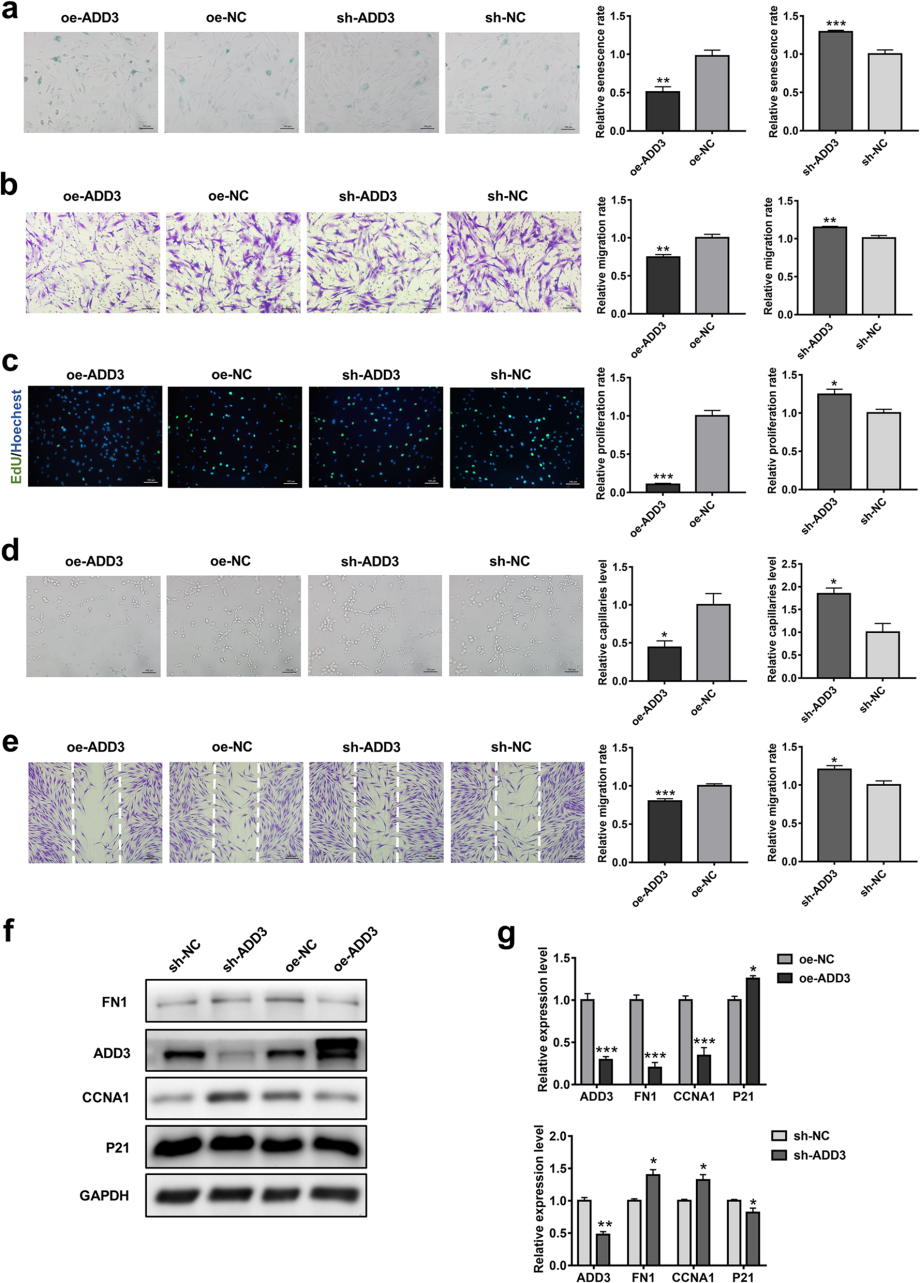
ADD3 endogenous expression and effect on ASCs. **a** β-gal staining analysis of senescent cells among ASCs transduced with empty vector (oe-NC), oe-ADD3, scramble small hairpin RNA (sh-NC), or sh-ADD3. Scale bar = 100 µm. **b** Representative images of migrated cells and quantitative analysis of ASCs that received the above treatment. Scale bar = 100 µm. **c** Representative images and quantitative analysis of EdU-stained cells (green) in ASCs that received the above treatment; nuclei were stained blue. Scale bar = 100 µm. **d** Images of tube formation and analysis of HUVECs treated with CM from ASCs that had been transduced with oe-NC, oe-ADD3, sh-NC, or sh-ADD3. Scale bar = 100 µm. **e** Images and analysis of migrated fibroblasts treated with CM as described above. Scale bar = 200 µm. **f** Western blotting analysis of the expression levels of ADD3, p21, FN1, and CCNA1 in ASCs transduced with oe-NC, oe-ADD3, sh-NC, or sh-ADD3. **g** Qualified data shown in (**f**). Data are shown as the mean ± SEM. All experiments were performed in triplicate. *n* = 3. *p < 0.05, **p < 0.01, ***p < 0.001 versus sh-NC group, #*p* < 0.05, ##*p* < 0.01, ###*p* < 0.001 versus oe-NC group

As a downstream gene of miR-143-3p, we presumed that ADD3 might be associated with ASC senescence. Notably, western blotting results indicated that protein expression levels of ADD3 and p21 were elevated in O-ASCs (Fig. 5j, k). Similar results were observed in replicative senescent ASCs (Fig. 5h, i). After transduction of the respective ASCs with either sh-ADD3 or oe-ADD3 lentiviral vector and the corresponding empty vector, substantial reduction or enhancement of ADD3 protein expression was observed (Additional file 1: Fig. S9). Cell proliferation and migration were slightly improved in ADD3 knockdown ASCs (Fig. 6b, c); SA-β-gal staining assays also indicated reduced cellular senescence in the sh-ADD3 group (Fig. 6a). Conversely, significant reduction of cellular function and enhancement of senescence were detected in ADD3-overexpressing ASCs, compared with NC-overexpressing ASCs. Consistent with the cell phenotype findings, functional proteins (e.g., FN1, CCNA1, and p21) were influenced by changes in ADD3 protein expression (Fig. 6f, g). Additionally, CM from ADD3-overexpressing ASCs or ADD3-deficient ASCs was compared with CM from control ASCs in terms of the abilities to induce angiogenesis and fibroblast migration. Importantly, CM from the ADD3-overexpressing group showed inferior abilities to induce angiogenesis and fibroblast migration, compared with the control group, whereas CM from ADD3-deficient ASCs showed superior abilities to induce these processes (Fig. 6d, e).

### ADD3 downregulation contributed to the anti-senescence effects of miR-143-3p in ASCs

**Fig. 7:**
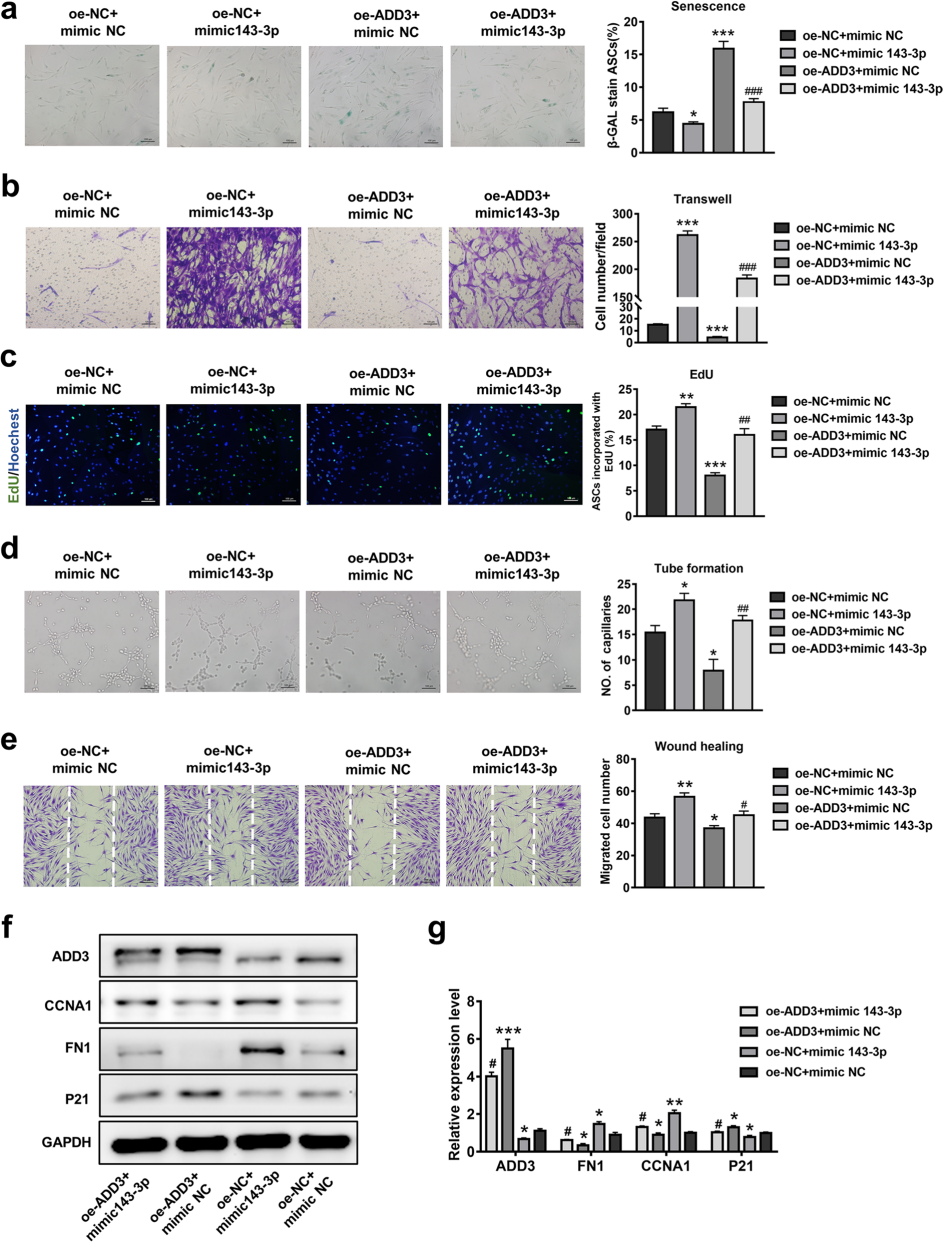
Effects of miR-143-3p and ADD3 on ASC senescence and cellular function. **a** β-gal staining and quantitative analysis of SA-β-gal-positive cells were performed in ASCs that had been stably transduced with empty vector (oe-NC) or ADD3 (oe-ADD3) and simultaneously transfected with miR-143-3p mimic or mimic-NC. Scale bar = 100 µm. **b** Images of migrated cells and quantitative analysis of ASCs that had been stably transduced with empty vector (oe-NC) or ADD3 (oe-ADD3) and simultaneously transfected with miR-143-3p mimic or mimic-NC. Scale bar = 100 µm. **c** Representative images and quantitative analysis of EdU-stained cells (red) in ASCs that received the above treatment; nuclei were stained blue. Scale bar = 100 µm. **d** Images of tube formation and analysis of HUVECs treated with CM from ASCs in the above four groups. Scale bar = 100 µm. **e** Images and analysis of migrated fibroblasts treated with CM as described above. Scale bar = 200 µm. **f** Western blotting analysis of the expression levels of p21, FN1, and CCNA1 in ASCs transduced with NC vector or SAN and simultaneously transfected with miR-143-3p mimic or mimic-NC. **g** Qualified data shown in f. Data are shown as the mean ± SEM. All experiments were performed in triplicate. *n* = 3. *p < 0.05, **p < 0.01, ***p < 0.001 versus oe-NC + mimic-NC group, #*p* < 0.05, ##*p* < 0.01, ###*p* < 0.001 versus oe-SAN + mimic-NC group

To examine whether ADD3 can reverse the rejuvenation effects of miR-143-3p in ASCs, stem cells containing oe-ADD3 vector or empty vector (oe-NC) were transfected with miR-143-3p mimic and corresponding mimic-NC. They were then subjected to analyses of cellular senescence, cell proliferation, and migration, as well as CM angiogenesis-promoting and fibroblast migration-promoting effects. As shown in Fig. 7b and c, compared with the oe-NC + mimic-NC group, the migration and proliferation of ASCs were enhanced in the oe-NC + miR-143-3p mimic group, but reduced in the oe-ADD3 + mimic-NC group. Moreover, compared with the oe-ADD3 + mimic NC group, miR-143-3p mimic treatment rescued the inhibitory effects on ASC proliferation and migration in the oe-ADD3 + miR-143-3p mimic group. Additionally, cellular senescence was significantly enhanced in the oe-NC + miR-143-3p mimic group and reduced in the oe-ADD3 + mimic-NC group (Fig. 7a). Functional proteins (e.g., FN1, CCNA1, and p21) in those groups showed similar tendencies (Fig. 7f, g). Furthermore, miR-143-3p mimic treatment in ASCs reversed the inhibitory effects of ADD3 overexpression on modulatory functions in HUVECs and fibroblasts (Fig. 7d, e). The findings support the hypothesis that miR-143-3p ameliorates senescence in ASCs by downregulating ADD3 (Additional file 4).

### Transplantation of SAN-depleted aged ASCs accelerates cutaneous wound healing in rats

**Fig. 8:**
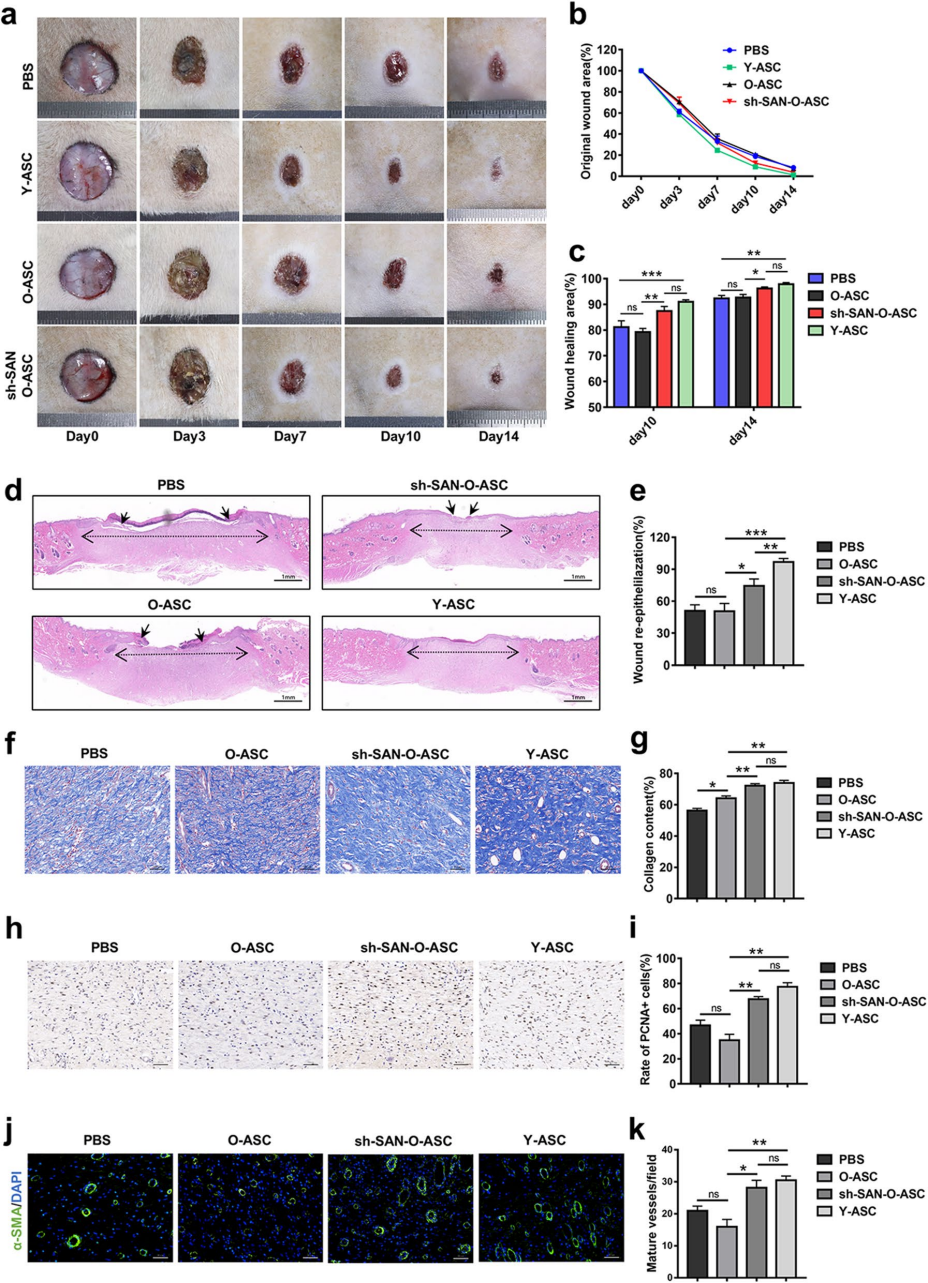
SAN-depleted O-ASCs promote wound repair capacity in vivo. **a** Representative images of wounds treated with PBS, Y-ASCs, O-ASCs, or sh-SAN-O-ASCs at different time points (*n* = 5). Images include a scale bar (ruler with basic unit of 1 mm). **b** Measurement of wound areas shown in (**a**). **c** Quantification of wound areas at 10- and 14-days post-wounding. **d** Representative hematoxylin and eosin staining analysis of skin tissues in each group at day 14 post-wounding. Single-headed arrows indicate the edge of un-epithelialized areas. Double-headed arrows indicate the edge of granulation tissue. Scale bar = 1 mm. **e** Qualification of the epithelialized areas shown in (**d**). **f** Representative Masson staining images and evaluation of collagen deposition in skin tissues in each group at day 14 post-wounding. Scale bar = 100 µm. **g** Qualification of the proportion of stained collagen shown in (**f**). **h** Immunohistochemical staining of PCNA expression in wound sections. Scale bar = 50 µm. **i** Enumeration and qualification of the ratio of PCNA-positive cells. **j** Immunofluorescent staining of skin tissues that received the above treatments at day 14 post-wounding. Smooth muscle cells (α-smooth muscle actin) and cell nuclei (DAPI) were stained green and blue. Scale bar = 50 µm. **k** Enumeration and qualification of mature vessels stained green. Scale bar, 100 μm. *n* = 4. *p < 0.05, **p < 0.01, ***p < 0.001. ns, not significant

To evaluate the rejuvenation and therapeutic effects of SAN-deficient ASCs on wound healing in vivo, full-thickness cutaneous wounds were created on the dorsal skin of rats. The edge of the wound area was treated with PBS, control vector-transduced Y-ASCs, control vector-transduced O-ASCs, or SAN-depleted old ASCs (sh-SAN-O-ASCs); wound healing status was then assessed. As shown in Fig. 8a–c, in contrast with the O-ASC group, the original wound area was significantly smaller in the sh-SAN-O-ASC group at days 7, 10, and 14 post-wounding. Furthermore, cutaneous wounds treated with sh-SAN-O-ASCs were completely healed at approximately 14 days post-wounding, similar to the results in the Y-ASC group.

Wound histological analysis was conducted to assess repair efficacy in each group at day 14 post-wounding. Hematoxylin and eosin staining showed that the sh-SAN-O-ASC group achieved greater re-epithelialization, compared with the O-ASC group (Fig. 8d, e); the mean proportions in the Y-ASC and PBS groups were 97% and 51%, respectively. Analysis of Masson’s trichrome-stained tissue showed that collagen deposition was greater in the sh-SAN-O-ASC group than in the O-ASC group; it was similar to the deposition in the Y-ASC group (Fig. 8f, g). Additionally, analysis of frozen skin sections showed that the survival rate was 2.5-fold greater in the sh-SAN-O-ASC group than in the O-ASC group (Additional file 1: Fig. S10).

Immunohistochemical and immunofluorescence analyses of wound tissue sections were performed to investigate the abilities of ASCs in each group to promote angiogenesis and cellular proliferation. Compared with the O-ASC group, the sh-SAN-O-ASC group exhibited significantly enhanced cellular proliferation ability, as determined by the proportion of PCNA-positive cells (Fig. 8h, i). Moreover, α-smooth muscle actin staining revealed approximately twofold more mature blood vessels in the sh-SAN-O-ASC group than in the O-ASC group (Fig. 8j, k). Collectively, these results indicated that SAN knockdown in aged ASCs was able to restore cellular therapeutic capabilities such as neovascularization and cellular proliferation in a cutaneous wound model.

## Discussion

Senescent ASCs generally show transcriptome variations and diminished therapeutic capacities, as demonstrated in our study. To our knowledge, this is the first study to investigate the lncRNA, SAN, which orchestrates aging-related ASC functions. SAN regulates the proliferation, migration, and cellular senescence of ASCs, as well as the effects of ASCs on fibroblasts and HUVECs. Mechanistic analyses showed that SAN acted as a competing endogenous RNA for miR-143-3p, which promotes ASC functions and inhibits senescence. Furthermore, miR-143-3p targets ADD3, thus controlling senescence in ASCs. SAN silencing reversed the aging process in ASCs and enhance the therapeutic potential of ASCs in wound healing, thereby accelerating wound healing and regeneration. This study demonstrated that the SAN–miR-143-3p–ADD3 pathway plays a key role in ASC senescence and therapeutic capabilities.

In the past few decades, stem cell-based therapies have been investigated by many researchers, autologous fat tissue transplantation have been safely used in the breast construction [30, 31]. The stromal vascular fraction (SVF), which is harvested by enzymatic digestion of subcutaneous adipose tissue, contains numerous ASCs [32]. The stromal vascular fraction has been used for the treatment of tissue defects in patients [33]. Mesenchymal stem cells have also been considered as a potential therapy for coronavirus disease 2019 [34, 35]. In the field of wound healing, ASCs enhance angiogenesis, reduce inflammation, accelerate wound closure and extracellular matrix remodeling, and promote skin regeneration [36]; thus, ASCs are considered ideal for clinical treatment [37]. However, negative factors such as donor age affect the potency and efficacy of ASCs [38], which limits their therapeutic applications. In this study, aged ASCs obtained from old volunteer donors showed impairments in the induction of angiogenesis and fibroblast migration in vitro, consistent with previous findings regarding senescent mouse ASCs used for in vivo wound healing analyses [4]. Various novel strategies have been proposed to rejuvenate aged stem cells; these include gene modification [39, 40], molecular treatment [41, 42], and noncoding RNA regulation [11, 20, 43]. Here, we confirmed that depletion of the lncRNA, SAN, can restore the function of aged ASCs in vitro and effectively promote cell survival, re-epithelialization, collagen deposition, and angiogenesis in vivo.

Multiple lncRNAs are reportedly involved in the regulation of cellular senescence [44]. The lncRNA, PANDA, interacts with scaffold-attachment-factor A to modulate the transcription of senescence-associated genes [45]. Bianchessi et al. [46] found that the mitochondrial lncRNA, ASncmtRNA-2, could act as a miRNA precursor and participate in endothelial cell replicative senescence and aging. In this study, we demonstrated that the lncRNA, SAN, was highly expressed in O-ASCs from old donor volunteers. Overexpression of SAN inhibited the proliferation and migration of ASCs, as well as their angiogenesis-promoting and fibroblast migration-promoting effects; it also enhanced cellular senescence. In contrast, SAN silencing ameliorated the senescent phenotype and restored the therapeutic potential of O-ASCs in a wound healing model, suggesting that SAN has a regulatory role in ASC aging. However, the mechanisms by which SAN participates in the regulation of mesenchymal stem cell senescence require further investigation. The broad function of cytoplasmic lncRNAs is the function of competing endogenous RNA. Through their primary sequence and secondary structure, lncRNAs can sequester miRNAs and serve as sponges for miRNAs [47]; thus, they can restrain the repression effect of miRNA on target genes and modulate the related cellular processes of miRNAs (e.g., cell differentiation and senescence) [16, 48]. We observed that SAN is mostly located in the cytoplasm and carries a binding site for miR-143-3p. Dual-luciferase reporter assays demonstrated that miR-143-3p can bind to SAN; the overexpression of miR-143-3p also reduced the expression of SAN and partly reversed the SAN-related functional decline in ASCs.

Mesenchymal stem cell senescence involves heterogeneous variations of transcripts including miRNAs, which have highly conserved sequences and play important regulatory roles in mesenchymal stem cell senescence [11, 48]. miRNAs reportedly modulate telomerase reverse transcriptase [49], transcription factors [43], mitochondrial fusion [22], and target mRNA translation [50], thereby influencing mesenchymal stem cell senescence phenotype and capacity. Importantly, downregulation of miR-143-3p has been detected in aged satellite cells and associated with myoblast senescence [23]. Similarly, we found that miR-143-3p expression was reduced in aged human ASCs, implying that the overexpression of miR-143-3p can enhance ASC functions (e.g., proliferation and migration) while ameliorating cellular senescence in ASCs. Thus, miR-143-3p presumably accelerates ASC senescence, whereas the inhibition of miR-143-3p rejuvenates ASCs.

miR‐143 regulates chamber morphogenesis by targeting and repressing ADD3 [51], suggesting that ADD3 is a target of miR-143-3p. In the present study, dual-luciferase reporter assays demonstrated that miR-143-3p can directly target the 3′-UTR of ADD3. ADD3 encodes cytoskeleton protein [52] and is generally regarded as an anti-oncogene [25], associated with biliary atresia [53]. Thus far, there have been no reports of ADD3 in mesenchymal stem cells. Our findings showed that ADD3 expression was significantly enhanced in senescent ASCs from old volunteer donors, which undergo replicative senescence. Moreover, ADD3 knockdown promoted ASC proliferation and migration, while inhibiting ASC senescence. The angiogenesis-promoting effect of CM derived from ADD3-silenced ASCs was consistent with the effect of CM from ADD3-deficient glioblastoma multiforme cells, reported by Kiang [26]. Furthermore, ADD3 silencing enhanced the migration-promoting effect of ASCs. In contrast, ADD3 overexpression enhanced cellular senescence and led to diminished cellular function. These results indicated that ADD3 is associated with ASC senescence and biological behavior as a target of miR-143-3p.

There were some limitations in this study. First, we focused on the role of the lncRNA, SAN, in restoring aged ASC function; however, we did not investigate whether the expression of SAN could regulate ASC differentiation in vitro or in vivo. Second, we did not investigate how functional molecules related to cell cycle, migration, and senescence were affected by ADD3. Finally, we did not investigate how other differentially expressed lncRNAs, miRNAs, or targets of miR-143-3p contributed to ASC senescence. Thus, further investigations are needed to address these points.

## Conclusion

In this study, we demonstrated that the aging-associated lncRNA, SAN, mediated age-related dysfunction in ASCs. Because the SAN–miR-143-3p–ADD3 axis plays an important role in regulating ASC senescence and therapeutic potency, our findings provide a potential target for rejuvenation of aged ASCs that may aid in stem cell-based transplantation treatment. Moreover, the findings imply that lncRNAs might be useful indicators of therapeutic capacity or degree of aging in ASCs.

### Supplementary Information

**Additional file 1**. **Table S1**: Basic characteristics of donors in the study. **Table S2**: Primers used for real-time polymerase chain reaction. **Table S3**: Sequences of mimics, and inhibitors. **Table S4**: Target sequences of lentiviral vectors containing shRNA. **Table S5**: Gene-specific primers used for the RACE analysis of NONHSAT035482.2. **Fig. S1**: Identification of fibroblast. **Fig. S2**: Evaluation of the cellular function of ASCs obtained from old and young volunteer donors. **Fig. S3**: Evaluation of the cellular senescence and proliferation of ASCs obtained from old and young volunteer donors. **Fig. S4**: Sequence structure of NONHSAT035482.2. **Fig. S5**: Examination of sh-SAN and oe-SAN vector efficiencies. **Fig. S6**: Evaluation of the cellular function of ASCs transduced with SAN mutant vector, SAN wild type vector or NC vector. **Fig. S7**: Examination of mimic and inhibitor transfection efficiencies. **Fig. S8**: SAN knockdown or overexpression can reduce or accelerate ADD3 expression. **Fig. S9**: Examination of sh-ADD3 and oe-ADD3 vector efficiencies. **Fig. S10**: SAN knockdown promotes ASC survival.

**Additional file 2**. Differentially expressed lncRNAs.

**Additional file 3**. Gene list in Fig. 5a.

**Additional file 4**. Original blots.

## Data Availability

All data generated and/or analyzed during this study are available from the corresponding author upon reasonable request. The RNA expression profiles were retrieved from the Gene Expression Omnibus (GEO) database (https://www.ncbi.nlm.nih.gov/geo/). The GEO series accession number is GSE174502.

## Abbreviations

ADAMTS9-AS2: ADAM metallopeptidase with thrombospondin type 1 motif 9 antisense RNA 2
ADD3: γ-Adducin
ASCs: Adipose stem cells
CCNA1: Cyclin A1
CM: Conditioned medium
DAPI: 4′,6-Diamidino2-phenylindole
FN1: Fibronectin 1
GAPDH: Glyceraldehyde-3-phosphate dehydrogenase
HCG11: HLA complex group 11
HUVECs: Human umbilical vein endothelial cells
lncRNAs: Long non-coding RNAs
MALAT1: Metastasis associated lung adenocarcinoma transcript 1
miRNAs: MicroRNAs
MUT: Mutant type
NcRNA: Non-coding RNA
OCT: Optical coherence tomography
P21: Cyclin dependent kinase inhibitor 1A
PBS: Phosphate-buffered saline
PCNA: Proliferating cell nuclear antigen
QPCR: Quantitative polymerase chain reaction
SAN: Senescence-associated non-coding RNA
SNHG5: Small nucleolar RNA host gene 5
UTR: Untranslated region
WT: Wild type
