# Supplementary material for: Knockdown of long noncoding RNA SAN rejuvenates aged adipose-derived stem cells via miR-143-3p/ADD3 axis
Source: Stem Cell Res Ther. 2023 Aug 21;14:213. doi: 10.1186/s13287-023-03441-1 (PMC10441736; doi:10.1186/s13287-023-03441-1)
Supplement: Supplementary file 1 — Additional file 1. Table S1: Basic characteristics of donors in the study. Table S2: Primers used for real-time polymerase chain reaction. Table S3: Sequences of mimics, and inhibitors. Table S4: Target sequences of lentiviral vectors containing shRNA. Table S5: Gene-specific primers used for the RACE analysis of NONHSAT035482.2. Fig. S1: Identification of fibroblast. Fig. S2: Evaluation of the cellular function of ASCs obtained from old and young volunteer donors. Fig. S3: Evaluation of the cellular senescence and proliferation of ASCs obtained from old and young volunteer donors. Fig. S4: Sequence structure of NONHSAT035482.2. Fig. S5: Examination of sh-SAN and oe-SAN vector efficiencies. Fig. S6: Evaluation of the cellular function of ASCs transduced with SAN mutant vector, SAN wild type vector or NC vector. Fig. S7: Examination of mimic and inhibitor transfection efficiencies. Fig. S8: SAN knockdown or overexpression can reduce or accelerate ADD3 expression. Fig. S9: Examination of sh-ADD3 and oe-ADD3 vector efficiencies. Fig. S10: SAN knockdown promotes ASC survival. [file 13287_2023_3441_MOESM1_ESM.pdf]

Table S1. Basic characteristics of different groups included in the study.

Variables	Young donators	Old donators	P value (Y/O)
Number	14	15	NA
Age	8.14 ± 2.82	54.20 ± 3.82	<0.0001****
Prefemoral fat	11	11	NA
Abdominal fat	3	4	NA
BMI	19.13 ± 3.57	22.02 ± 2.61	0.04*
SBP	115 ± 11.67	126.54 ± 8.30	0.03*
DBP	75 ± 7.39	80 ± 6.15	0.14
FBG	4.87 ± 0.35	5.00 ± 0.69	0.60
TG	1.15 ± 0.43	1.53 ± 0.46	0.29
TC	3.68 ± 0.53	4.44 ± 0.80	0.20
HDL	1.23 ± 0.18	1.19 ± 0.31	0.83
LDL	1.98 ± 0.55	2.65 ± 0.71	0.01*
Cr	36.5 ± 5.00	51.08 ± 16.26	0.02*
BUN	3.89 ± 1.16	4.13 ± 0.87	0.40

BMI (kg/m^2): body mass index; SBP (mm Hg): systolic blood pressure; DBP (mm Hg): diastolic blood pressure; FBG (mmol/L): fasting blood glucose; TG (mmol/L): triglyceride; TC (mmol/L): total cholesterol; HDL-C (mmol/L): high-density lipoprotein cholesterol; LDL-C (mmol/L): low-density lipoprotein cholesterol; Cr (μmol/L): creatinine; BUN (mmol/L): blood urea nitrogen. Data are expressed as Mean ± SEM and were statistically analyzed by the un-paired Student's t test. *p< 0.05, **p< 0.01.

Table S2. RNAs' primers used for real-time polymerase chain reaction.

Gene	Primer sequences
NONHSAT035482.2	Forward 5'-CGCAGTCTTGGATGATGGGTTC-3'
	Reverse 5'-GCTCCAGCTACTTTGGGGCTTA-3'
ADD3	Forward 5'-TTCACCTCCTCTCAGTCTTGGC-3'
	Reverse 5'- GTGTGCCCATCCAAACAAGTC-3'
VEGFA	Forward 5'- GTTCGAGGAAAGGGAAAGGGGC-3'
	Reverse 5'- TGAGCAAGGCCCACAGGGAAC-3'
FGF2	Forward 5'- GAGAAGAGCGACCCTCACATCA-3'
	Reverse 5'- TGCCCAGTTCGTTTCAGTGCC-3'
U3	Forward 5'- TGTAGAGCACCGAAAACCACG-3'
	Reverse 5'- CAGCCAAGCAACGCCAGA -3'
GAPDH	Forward 5'- AAAATCAAGTGGGGCGATGCT-3'
	Reverse 5'- TGGTTCACACCCATGACGAAC-3'
β-ACTIN	Forward 5'-CAGCCTTCCTTCCTGGGCAT-3'
	Reverse 5'-GGGCAGTGATCTCCTTCTGCAT-3'
hsa-miR-143-3p	Forward 5'- TGAGATGAAGCACTGTAGCTC-3"
U6	Forward 5'- CTCGCTTCGGCAGCACA-3'
	Reverse 5'- AACGCTTCACGAATTTGCGT-3'

Table S3. Sequences of mimics, inhibitors.

	Sequences
hsa-miR-143-3p mimic	5'- UGAGAUGAAGCACUGUAGCUC -3"
	3'- ACUCUACUUCGUGACAUCGAG -5'
mimic NC	5'- UUUGUACUACACAAAAGUACUG -3'
	3'- AAACAUGAUGUGUUUUCAUGAC -5'
hsa-miR-143-3p inhibitor	5'- GAGCUACAGUGCUUCAUCUCA -3'
inhibitor NC	5'- CAGUACUUUUGUGUAGUACAAA-3'

Table S4. Target sequences of lentiviral vector containing shRNA.

Gene	Target sequences
sh-SAN#1	5’- GCTCTCAACATCTCTTCTT -3’
sh-SAN#2	5’- GCAGGAACTTGATTACTTT -3’
sh-SAN#3	5’- GCAACTTTGTTCAACATTA -3’
sh-ADD3#1	5’- GCTGTATCCTCCATGAAAT -3’
sh-ADD3#2	5’- GCCACCTTCTACTATGCAA -3’
sh-ADD3#3	5’- GCTCCTCCTAACCCATTTA -3’
sh-NC	5’- CCTAAGGTTAAGTCGCCCTCG -3’

Table S5. The gene-specific primers used for the RACE analysis of NONHSAT035482.2.

Gene Specific Primers	Sequences
5'RACE GSP	
Out Primer	5’- ACAGTGGCAGAAGAAGTCCAGCAAGG -3”
Inner Primer	5’- GGAACCCATCATCCAAGACTGCGGCAC -3”
3'RACE GSP	
Out Primer	5’- AGGAGGCAGGTCTCCGCGGTTCATCT -3”
Inner Primer	5’- TGCCGCAGTCTTGGATGATGGGTTCC -3”

Figure S1

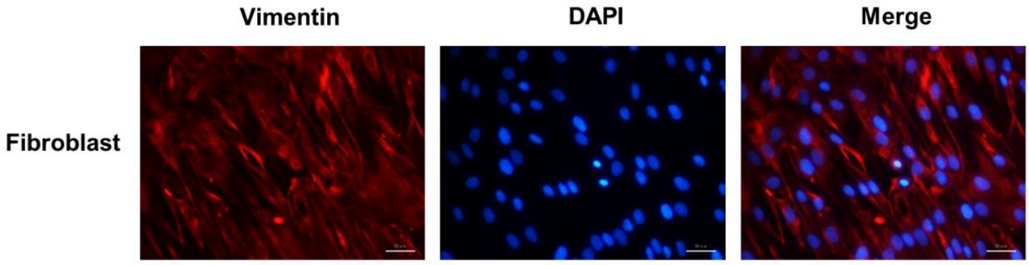

Figure S1. Identification of fibroblast. Images of vimentin-stained cells (red) in fibroblast; nuclei were stained blue. Scale bar = 50 μm.

Figure S2

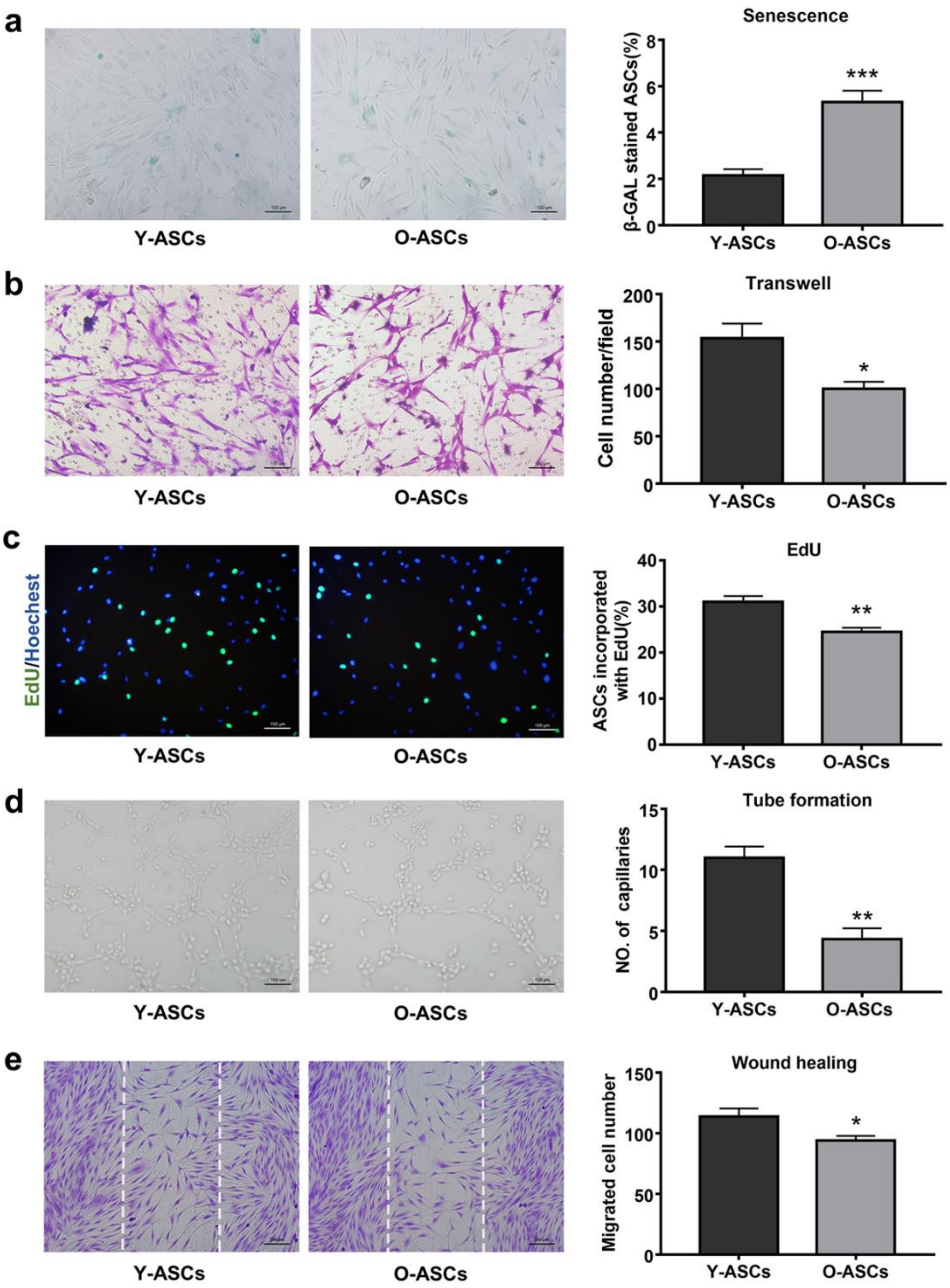

Figure S2. Evaluation of the cellular function of ASCs obtained from old and young volunteer donors. (a) Representative images of SA-β-gal staining and quantitative analysis of SA-β-gal-positive cells in Y-ASCs and O-ASCs. Scale bar = 100 μm. (b) Images and quantitative analysis of migrated cells in Y-ASCs and O-ASCs. Scale bar = 100 μm. (c) Representative images of EdU-stained cells (green) in ASCs and analysis of proliferative rate in those two groups; nuclei were stained blue. Scale bar = 100 μm. (d) Images of tube formation and quantitative analysis of capillaries in HUVECs treated with CM from Y-ASCs and O-ASCs. Scale bar = 100 μm. (e) Images and quantitative analysis of migrated fibroblasts that received the above treatments. Scale bar = 200 μm. All experiments were performed in triplicate (n = 5. $*p < 0.05$, $**p < 0.01$, $***p < 0.001$).

Figure S3

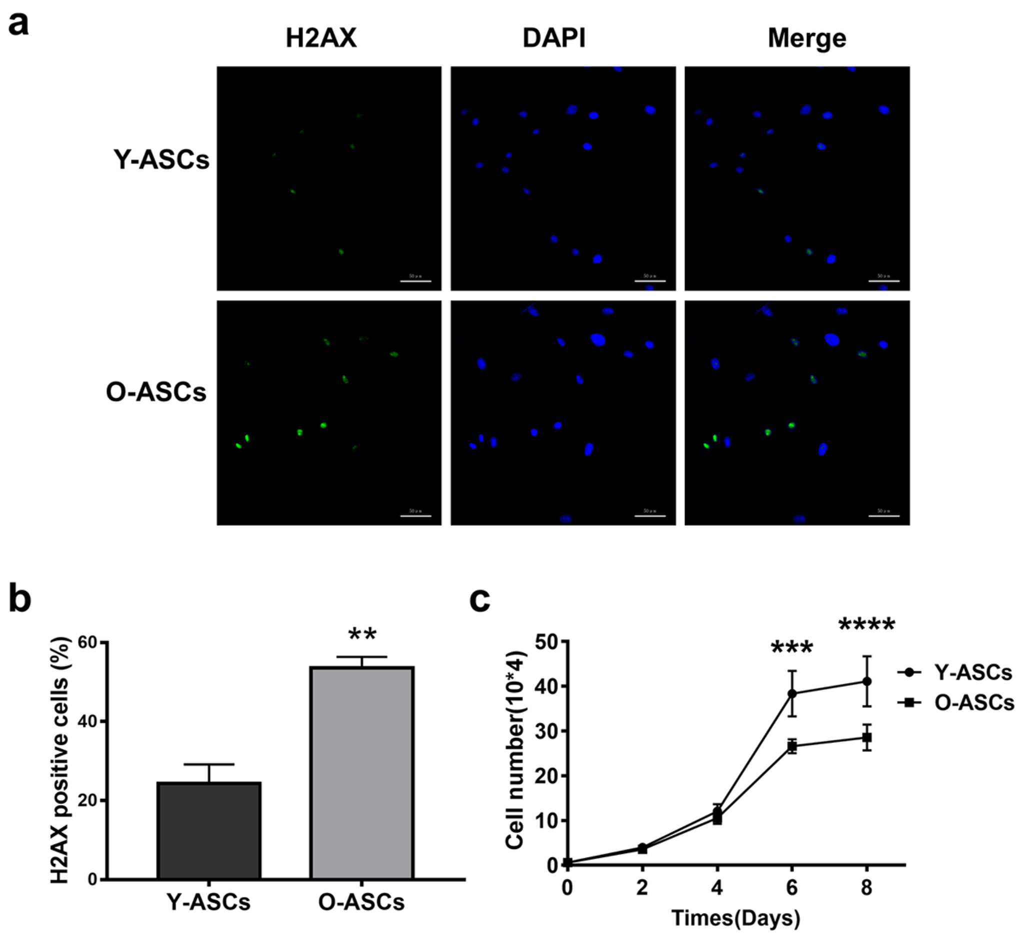

Figure S3. Evaluation of the cellular senescence and proliferation of ASCs obtained from old and young volunteer donors. (a) Representative images of H2AX stained cells (green) in Y-ASCs and O-ASCs; nuclei were stained blue. (b) Quantitative analysis of H2AX positive cells rates in Y-ASCs and O-ASCs. (c) Cell growth curves of O-ASCs and Y-ASCs. Scale bar = 50 μm. All experiments were performed in triplicate (n = 4. *$p < 0.05$, **$p < 0.01$, ***$p < 0.001$, ****$p < 0.0001$).

Figure S4

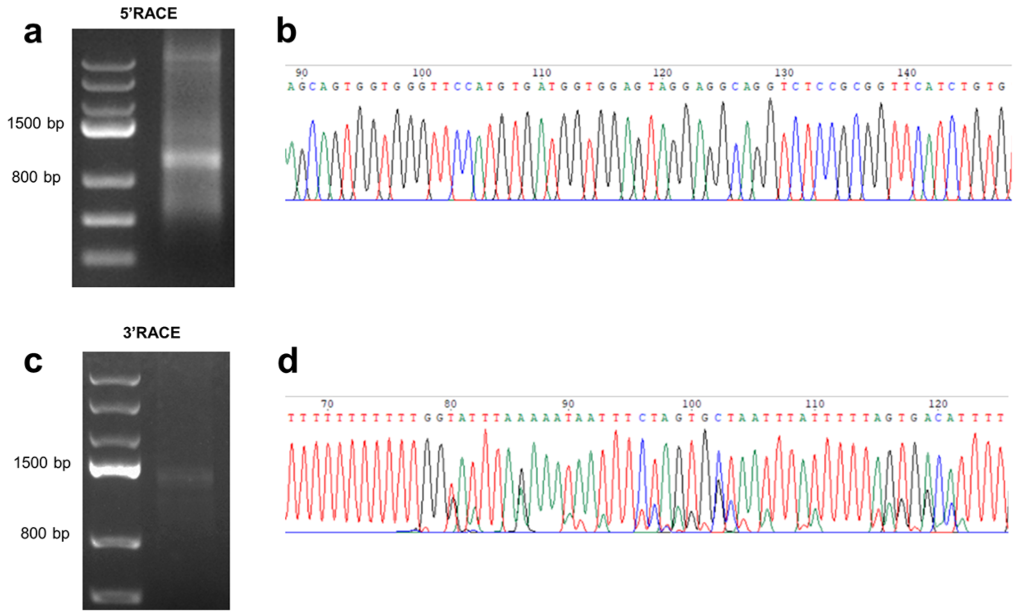

e

AGCAGTGGTGGGTTCCATGTGATGGTGGAGTAGGAGGCAGGTCTCCGCGGTTCATCTGTGTTGCTCTAAATGACACTGTTTCATTATTT
TGATGGCTGGAGAATATTTCCTAGTGTATGTATATGAGAGTTTCTTGATCTCTTTATCTGTGGATGAACAGCTACTTTGAAACATATGATAC
ATTTGTGTTAAGGCTAGTCACCCTGCTGTGGAATAGAAGGCCAGAATTGATCAGTCTCATCTGAGAGTAACTTTGTACCCATCACTGATT
CCTTCTGAGACTGCCTCCACTTCCCCAGCAGCCTCTGGTTTCTTCATGTGGCTGCAGATGGCAGGATTTCCCAAAGGTTTCTGGCTGA
AACATATTCCGTGGTGTATCTGTACAGCAGTTTCCTCATCCCTGCAGCTGTGTTTGAACAGGTCATTTACCATGCTGTCCTCCAGGTTCA
ACAGTATGGCTCCAAAGGATGAAATTTCATTCTGATTTTCTGGCTGAAGACTATTCTCTTTGTGTATGTCCACCACAGTTACTTTATCCCT
TCATCTGTGGATGGGCAGTCTCGCTGTATTGCCCAGGCTGGAGTGCAGTGGCATGATCTCAGCTCACTGCAAGCTCTGCTTCCTGGGT
TCACGCCATTCTCCTGCCTCAGCCTCCTGAGTAGCTGGGATTACAGGCACCCGCCACCACGCCCAGGAAACTCCAAACTGTCCAAGG
AGATAGTTCTGTTGTGATTACTTCATTGAGAAATTTAACTTATGAGCAGTTGAAAGGAATGCAAGTTGCTGCAAAATCAGAATGAAGAGT
GCAAAACGACTAAGCTACAATGTTTTGTCATTATTCACTCTGATGTGAAAAAGGCAGTGAATTTAATAGAAAATAACTTCGTAGAGCAAA
ATCTCAGGTGTGTTTTTTTAGTGCCGCAGTCTTGGATGATGGGTTCCTAGAAGCTCTCAACATCTCTTCTTAATTGGAGAAAGTGTTAAG
CCCCAAAGTAGCTGGAGCAGTACATCTTCAATTTTTGACAAGAAAGCAGGAACTTGATTACTTTGAGTGCTATTCATTAGTTTCTGCTTT
CATTGAGAATGCAACAAAAGCCAACTAGGCTGCTGCTAACTCCTTGCTGGACTTCTTCTGCCACTGTCACAGGAACTGTAATCTCACTG
GACAATTAACTAGGGAGTCTTTCATCTTGAGTGACTGCTGCACAAATGATCTTCAAAGCATTTTAGCCACCAGAGGAATTCTCTTGAAAT
ACCCAAAATCCATCAGTATCTTGAATCATGCTGGATTTTGAAGAATTCTTAACAAGCCATGTAAAGGGGGCTCTCTGGCCTTGAAATAGT
GATGTTTTTTATACAGAAAGGAGAATGCAGAATGGTCAGACTACCATGCACTGTTAAATTTGATTTCAAGAAATTACAGGAAAACTTTCC
AAAGTTCCATCTCACAGAAATTATTTTTACAAAGAATTCCAAGATAAGTTTAGTTTTATGGAAGACTTTTATGTGGTTTTTACTCACTCTTC
ATCTCAGACATCAACAGATGATTACATCACTTATTTAGCTAGTAAATTTATTAATATAAAAACTCAGAGACATTCCAATATCCACATTGCTT
ACACCATTAGGCATAGATTCAGTGTCAGCTATGACAATTGAAAATAAGCTGTTTTGTGATTTAAAGGTTTAAATTTCTCTAACCAAACTGC
TTGATCCAGATGCAGGACTGCAAATGTTAATATTTGTTCTGGAAGAACAATCAAATAAGACTTAAGAGGAAAAGGAATGGCCACAATCC
ACCTGAAATTTTTTTTTTAAAAAGTGTGCAGCCTACTAAATCAGAATGAAAATAGAAGTACAAGATTATAAACAAAATGCAATCAAACTTT
TCTTAAGCTTACCTAAAGTTATTTCATCTGAAAATTTCAAGCAACTTTGTTCAACATTAAATTGACAATCTAAACTAACAAGTCTTTTGAAT
TTATGCATGGTAGTAAACATTCTCTCTATTAACTTTATTACCTAAGGCTAAACCTAAAATTTTTAAGCAAAATTAGAAAAATAGTCTTCACT
CATCAAAAAAATAAAGTTTGTTACATTTAGTATTTTCCCAATAAAATTGGTCGTTCTTGGTTTTTTATTTGGAGAGTCTGTGCAAAATGTCAC
TAAAAATAAATTAGCACTAGAAATTATTTCTAAATACCAAAAAAAAAAAAAAATGAAGAATGGTTTCACAAAGAAAA

Figure S4. Sequence structure of NONHSAT035482.2. (a, b) 5'-RACE and 3'-RACE were conducted to harvest the 5'-end and 3'-end cDNA sequences of NONHSAT035482.2. (c, d) Amplicons were then sequenced. (e) The nucleotide sequence of the full-length NONHSAT035482.2 is shown red zone and underlined bases are the region of 5'-end (942 bp) and 3'-end (1301 bp) nucleotide sequences, respectively. A polyadenylation sequence was detected at the 3'-end of NONHSAT035482.2.

Figure S5

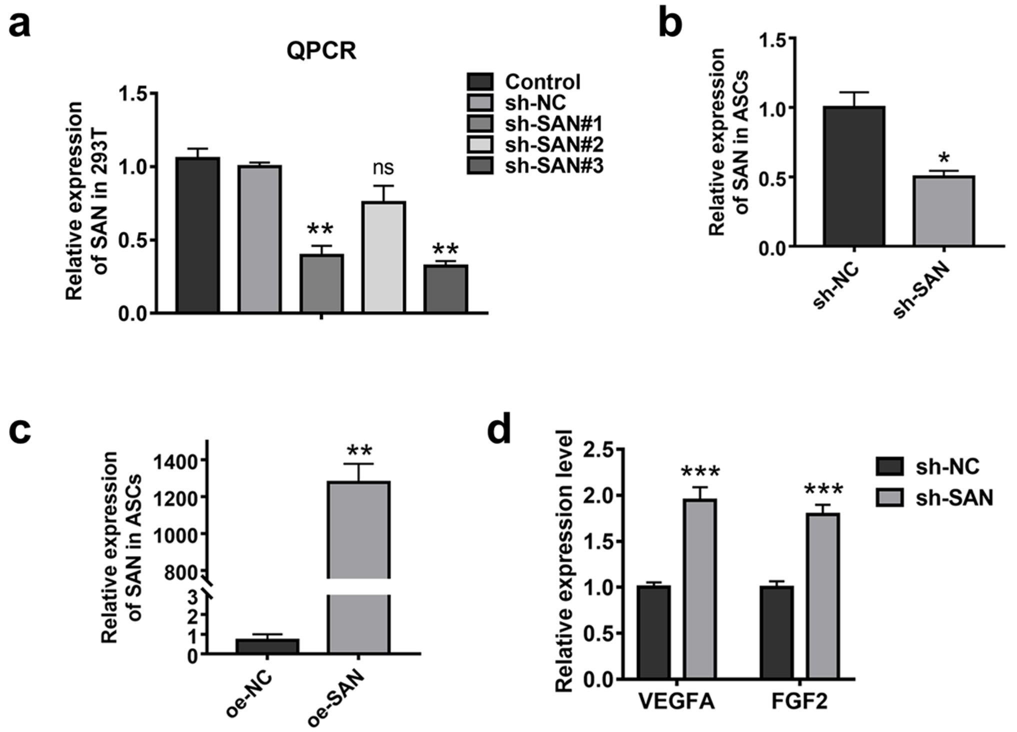

Figure S5. Examination of sh-SAN and oe-SAN vector efficiencies. (a) The expression of SAN in HEK-293T cells after transfection with sh-SAN plasmids. (b) qPCR analysis of SAN expression in ASCs transduced sh-SAN or sh-NC. (c) qPCR analysis of SAN expression in ASCs transduced oe-SAN or oe-NC. (d) qPCR analysis of the expression of vascular endothelial growth factor and fibroblast growth factor in ASCs transduced with sh-SAN or sh-NC. All experiments were performed in triplicate (n = 3. *p < 0.05, **p < 0.01, ***p < 0.001).

Figure S6

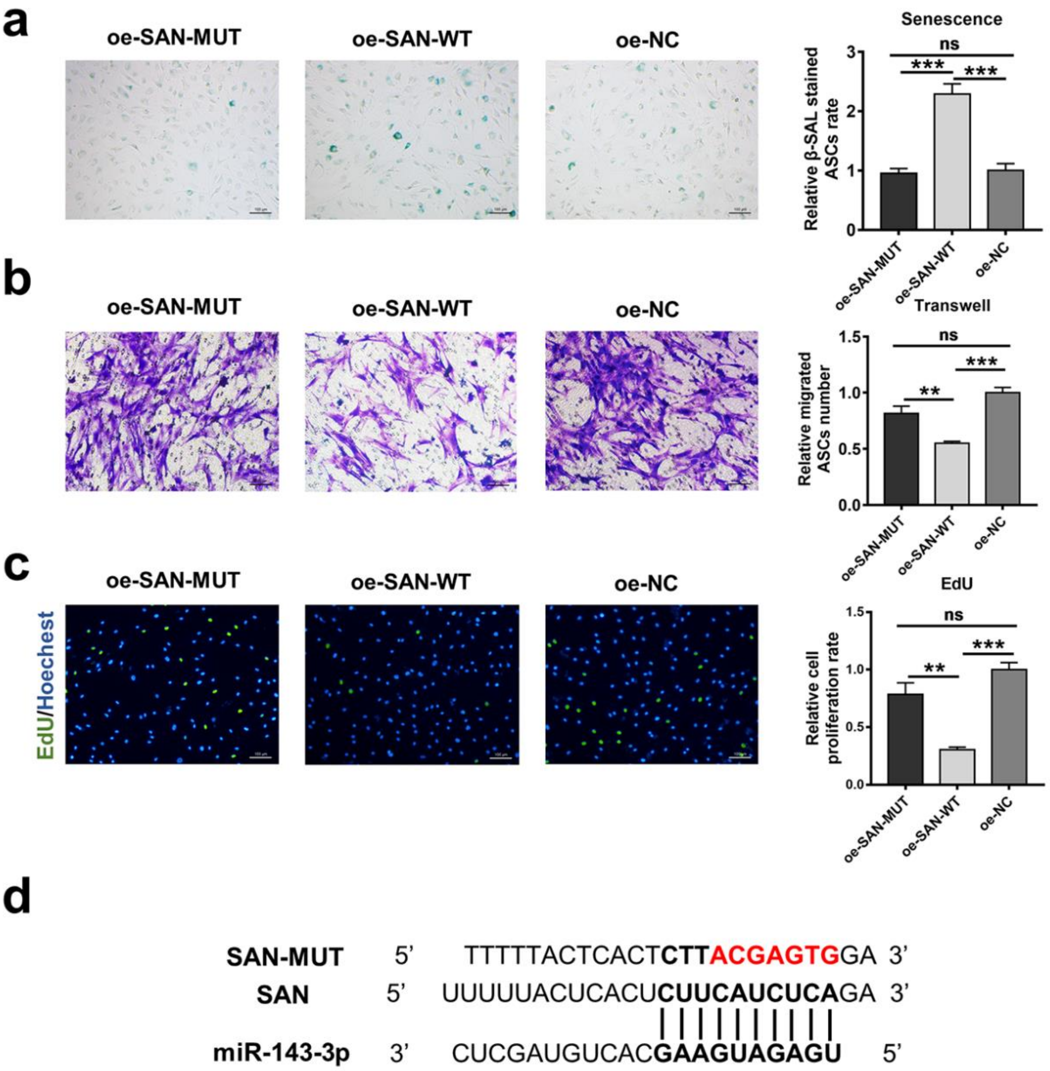

Figure S6. (a) β-gal staining and quantitative analysis of SA-β-gal-positive cells were detected in ASCs that had been stably transduced with SAN mutant type vector, SAN wild type or NC. Scale bar = 100 µm. (b) Images of migrated cells and quantitative analysis of ASCs that had been stably transduced with SAN mutant type vector, SAN wild type or NC. Scale bar = 100 µm. (c) Representative images and quantitative analysis of EdU-stained cells (green) in ASCs that received the above treatment; nuclei were stained blue. Scale bar = 100 µm. (d) Mutations in the sequence of SAN mutant type vector. All experiments were performed in triplicate (n = 3. *$p < 0.05$, **$p < 0.01$, ***$p < 0.001$).

Figure S7

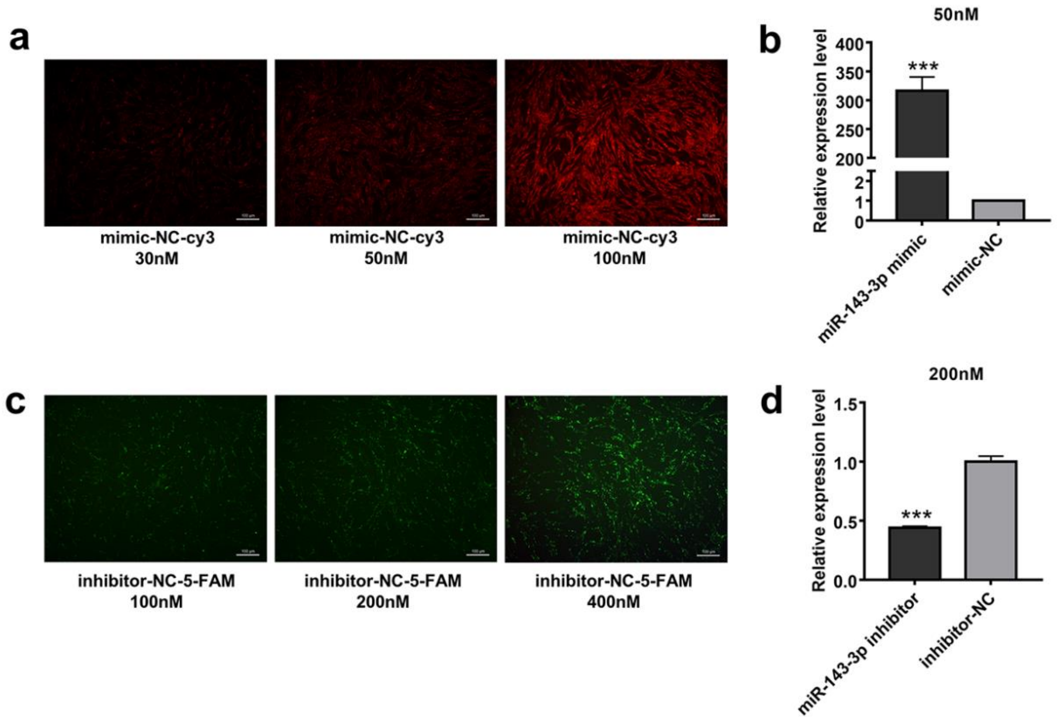

Figure S7. Examination of mimic and inhibitor transfection efficiencies. (a) Representative images of ASCs treated with mimic-NC labeled with cy3 (red) in different concentration. Scale bar = 100 μm. (b) qPCR was conducted to detect miR-143-3p expression in ASCs transfected with 50nM miR-143-3p mimic. (c) Representative images of ASCs treated with inhibitor-NC labeled with 5-FAM (green) in different concentration. Scale bar = 100 μm. (d) qPCR was conducted to detect miR-143-3p expression in ASCs transfected with 200nM miR-143-3p inhibitor. All experiments were performed in triplicate (n = 3. ***p < 0.001).

Figure S8

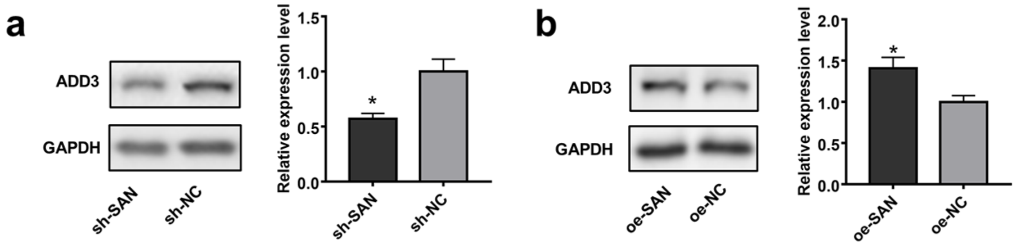

Figure S8. SAN knockdown or overexpression could reduce or accelerate ADD3 expression. (a) Western blotting and quantitative analysis of the expression levels of ADD3 in ASCs transduced with sh-SAN or sh-NC. (b) Western blotting and quantitative analysis of the expression levels of ADD3 in ASCs transduced with NC vector or SAN. All experiments were performed in triplicate (n = 3. *p < 0.05).

Figure S9

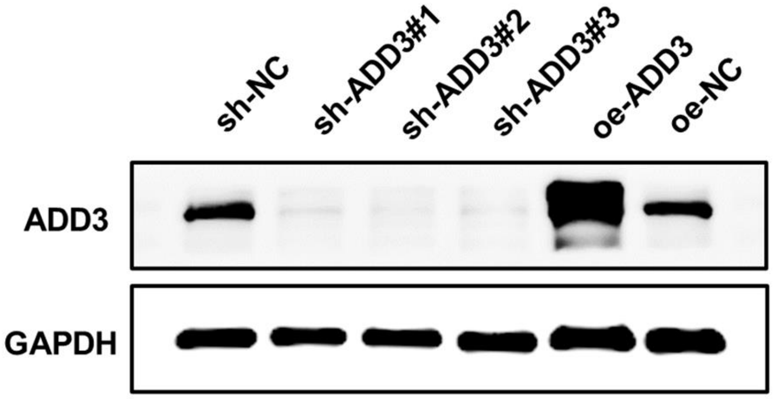

Figure S9. Examination of sh-ADD3 and oe-ADD3 vector efficiencies. Western blotting analysis of the expression levels of ADD3 in ASCs transduced with sh-ADD3 or sh-NC and oe-ADD3 or oe-NC.

Figure S10

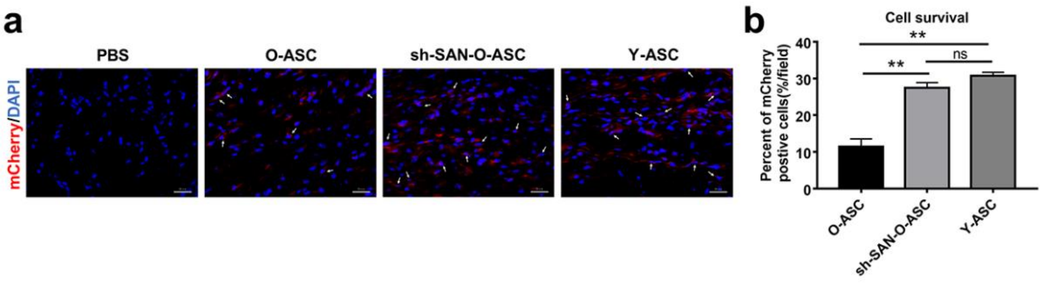

Figure S10. SAN knockdown promotes ASC survival. (a) Representative images of wound sections that underwent injection of PBS, Y-ASCs, O-ASCs, or sh-SAN-O-ASCs at day 14 post-wounding. Cell nuclei (DAPI) were stained blue. Survival of implanted cells was detected by mCherry (red) expression and DAPI (blue) staining in the subcutaneous region of the wound; non-specific fluorescent staining was excluded. Arrows indicate surviving ASCs. Scale bar, 50 µm. (b) Enumeration of surviving ASCs. All experiments were performed in triplicate (n = 4–5. **p < 0.01, ns, not significant).
