# Supplementary material for: Knockdown of long noncoding RNA SAN rejuvenates aged adipose-derived stem cells via miR-143-3p/ADD3 axis
Source: Stem Cell Res Ther. 2023 Aug 21;14:213. doi: 10.1186/s13287-023-03441-1 (PMC10441736; doi:10.1186/s13287-023-03441-1)

**Original blots: repeat 1 was used in formal figures**

## Three replicative experiments in Figure 1i&1j

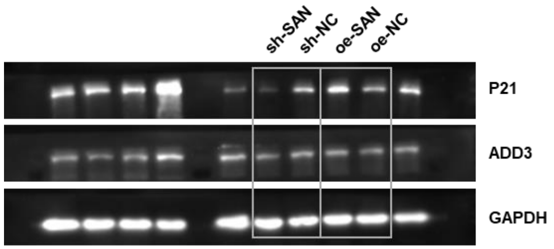


**Repeat 1**

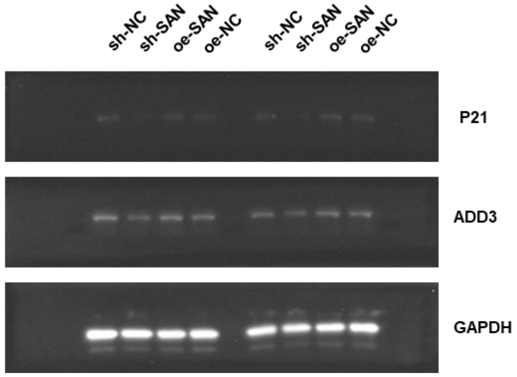


**Repeat 2** **Repeat 3**

## Three replicative experiments in Figure 3f

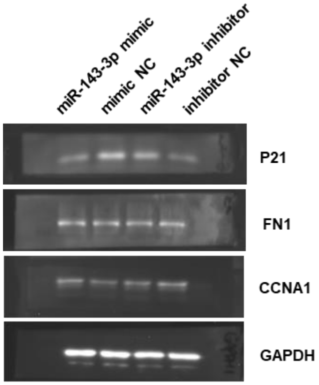


**Repeat 1**

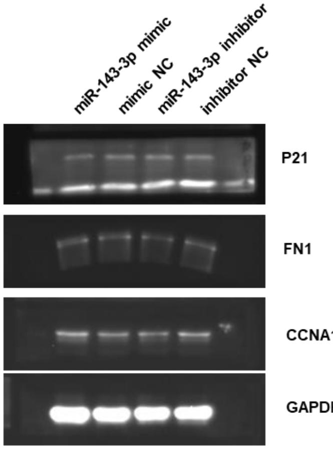


**Repeat 2**

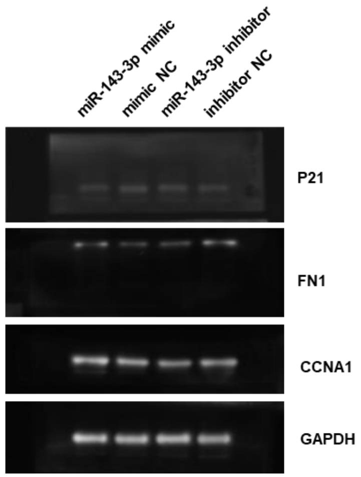


**Repeat 3**

## Three replicative experiments in Figure 4f

**Repeat 1**

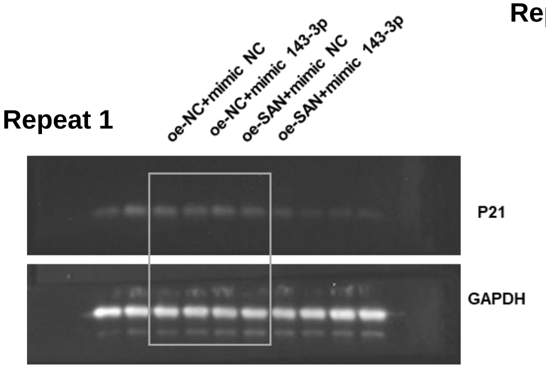


**Repeat 2** **Repeat 3**

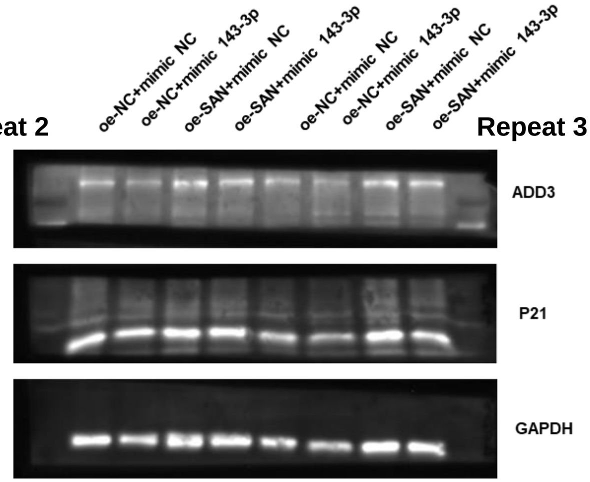

## Three replicative experiments in Figure 5g

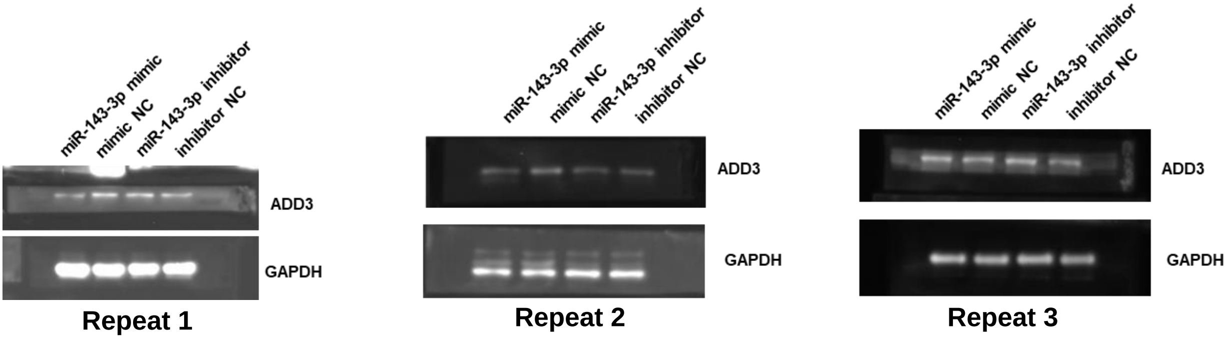


## Three replicative experiments in Figure 6f

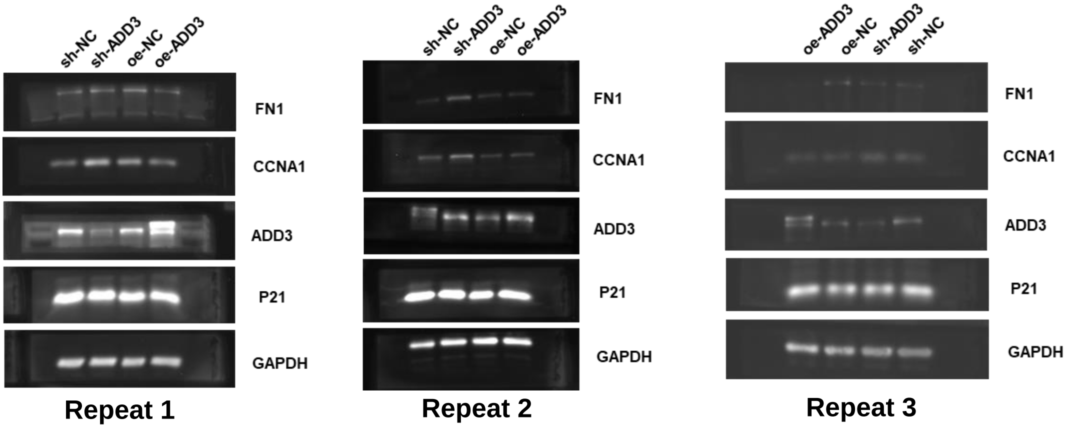


## Three replicative experiments in Figure 7f

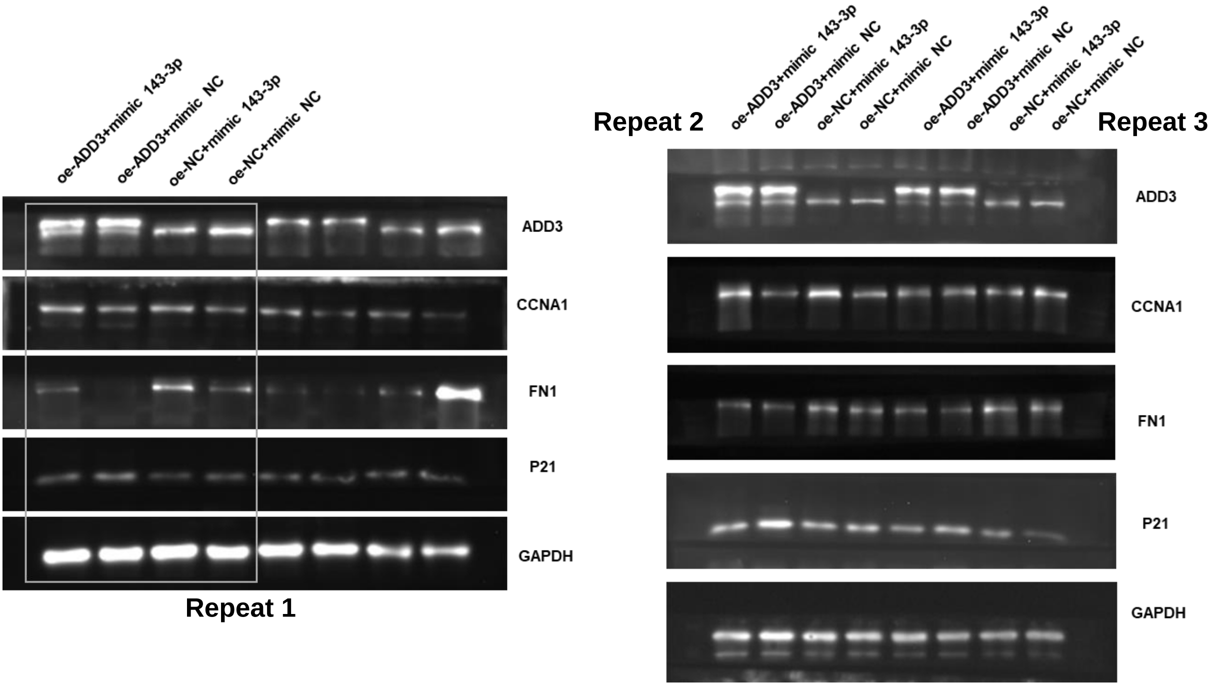

Figure 5h

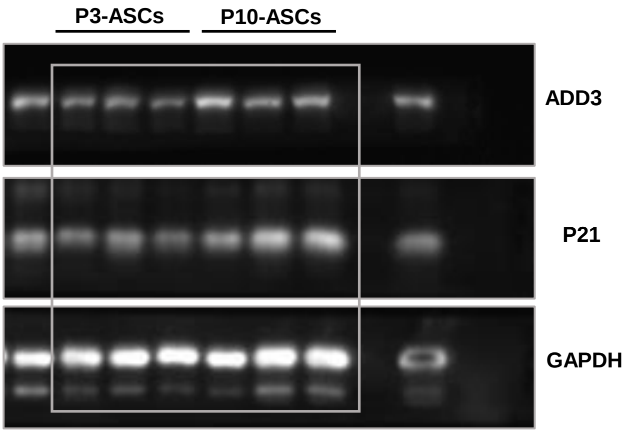


Figure 5j

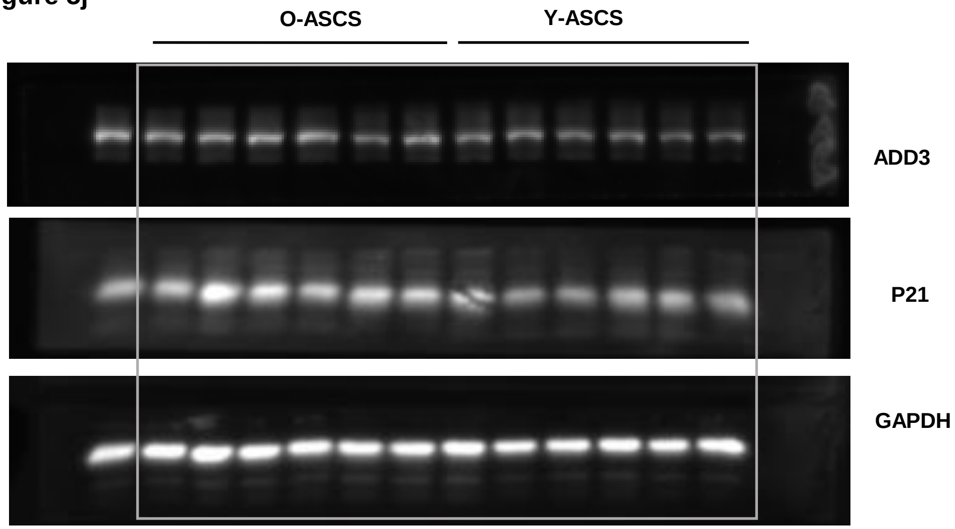

Supplement: Supplementary file 4 — Additional file 4. Original blots. [file 13287_2023_3441_MOESM4_ESM.pdf]
